# Supplementary material for: Complex scaffold remodeling in plant triterpene biosynthesis
Source: Science. Author manuscript; Available in PMC 2023 Mar 1. (PMC9976607; doi:10.1126/science.adf1017)
Supplement: Data S1 [file NIHMS1868721-supplement-Data_S1.docx]

Data S1- NMR spectra for all isolated compounds

[Figure 1. NMR spectra of apo-melianol (**3**) (*M. azedarach*).](#_heading=h.30j0zll) **2**

[Figure 2. NMR Assignment of (**6**) (*C. sinensis*).](#_heading=h.1fob9te) **3**

[Figure 3. ^1^H spectrum for (**6**) (*C. sinensis*).](#_heading=h.3znysh7) **4**

[Figure 4. ^13^C spectrum for (**6**) (*C. sinensis*).](#_heading=h.2et92p0) **5**

[Figure 5. COSY spectrum for (**6**) (*C. sinensis*).](#_heading=h.tyjcwt) **6**

[Figure 6. HSQC spectrum for (**6**) (*C. sinensis*).](#_heading=h.3dy6vkm) **7**

[Figure 7. HMBC spectrum for (**6**) (*C. sinensis*).](#_heading=h.1t3h5sf) **8**

[Figure 8. NMR Assignment of (**4’**) (*C. sinensis*).](#_heading=h.4d34og8) **9**

[Figure 9. ^1^H spectrum for (**4’**) (*C. sinensis*).](#_heading=h.2s8eyo1) **10**

[Figure 10. ^13^C spectrum for (**4’**) (*C. sinensis*).](#_heading=h.17dp8vu) **11**

[Figure 11. COSY spectrum for (**4’**) (*C. sinensis*).](#_heading=h.3rdcrjn) **12**

[Figure 12. HSQC spectrum for (**4’**) (*C. sinensis*).](#_heading=h.26in1rg) **13**

[Figure 13. HMBC spectrum for (**4’**) (*C. sinensis*).](#_heading=h.lnxbz9) **14**

[Figure 14. NMR spectra of 21(S)-acetoxyl-apo-melianone (**6**) (*M. azedarach*).](#_heading=h.35nkun2) **15**

[Figure 15. NMR assignment of degraded luvungin A (**7**) (*C. sinensis*).](#_heading=h.1ksv4uv) **16**

[Figure 16. ^1^H spectrum for degraded (**7**) (*C. sinensis*).](#_heading=h.44sinio) **17**

[Figure 17. ^13^C spectrum for degraded (**7**) (*C. sinensis*).](#_heading=h.2jxsxqh) **18**

[Figure 18. COSY spectrum for degraded (**7**) (*C. sinensis*).](#_heading=h.z337ya) **19**

[Figure 19. HSQC spectrum for degraded (**7**) (*C. sinensis*).](#_heading=h.3j2qqm3) **20**

[Figure 20. HMBC spectrum for degraded (**7**) (*C. sinensis*).](#_heading=h.1y810tw) **21**

[Figure 21. NMR assignment of 1-hydroxyl luvungin A (**9**) (*C. sinensis*).](#_heading=h.4i7ojhp) **22**

[Figure 22. ^1^H spectrum for (**9**) (*C. sinensi*s).](#_heading=h.2xcytpi) **23**

[Figure 23. ^13^C spectrum for (**9**) (*C. sinensis*).](#_heading=h.1ci93xb) **24**

[Figure 24. COSY spectrum for (**9**) (*C. sinensis*).](#_heading=h.3whwml4) **25**

[Figure 25. HSQC spectrum for (**9**) (*C. sinensis*).](#_heading=h.2bn6wsx) **26**

[Figure 26. HMBC spectrum for (**9**) (*C. sinensis*).](#_heading=h.qsh70q) **27**

[Figure 27. NMR spectra of epi-neemfruitin B (**10**) (*M. azedarach*).](#_heading=h.3as4poj) **28**

[Figure 28. NMR assignment of (**13**) (*C. sinensis*).](#_heading=h.lotd6s631at3) **29**

[Figure 29. ^1^H spectrum for (**13**) (*C. sinensis*).](#_heading=h.muznz678q4zg) **30**

[Figure 30. COSY spectrum of (**13**) (*C. sinensis*).](#_heading=h.7aq8tejgzpuj) **31**

[Figure 31. NMR assignment of (**13’**) (*C. sinensis*).](#_heading=h.rw1x7iacw1xi) **32**

[Figure 32. ^1^H spectrum for (**13’**) (*C. sinensis*).](#_heading=h.n7jarq3hpm9n) **33**

[Figure 33. ^13^C spectrum for (**13’**) (*C. sinensis*).](#_heading=h.hu5yz1i56a8i) **34**

[Figure 34. COSY spectrum for (**13’**) (*C. sinensis*).](#_heading=h.pcccsn9os7y4) **35**

[Figure 35. HSQC spectrum for (**13’**) (*C. sinensis*).](#_heading=h.14zbt4xtl3p1) **36**

[Figure 36. HMBC spectrum for (**13’**) (*C. sinensis*).](#_heading=h.mza71pomnq27) **37**

[Figure 37. NMR spectra of (**14**) (*M. azedarach*).](#_heading=h.1pxezwc) **38**

[Figure 38. Partial NMR assignment for kihadalactone A (**19**)](#_heading=h.49x2ik5)[(*C. sinensis*).](#_heading=h.mza71pomnq27) **39**

[Figure 39. ^1^H spectrum for kihadalactone A (**19**)](#_heading=h.2p2csry) [(*C. sinensis*).](#_heading=h.mza71pomnq27) **40**

[Figure 40. COSY spectrum for kihadalactone A (**19**)](#_heading=h.147n2zr) [(*C. sinensis*).](#_heading=h.mza71pomnq27) **41**

[Figure 41. NMR spectrum of (**20**) (*M. azedarach*).](#_heading=h.3o7alnk) **42**

[Figure 42. ^13^C NMR spectrum of analytical standard of azadirone (**18**).](#_heading=h.bwyhncnrpgt) **43**


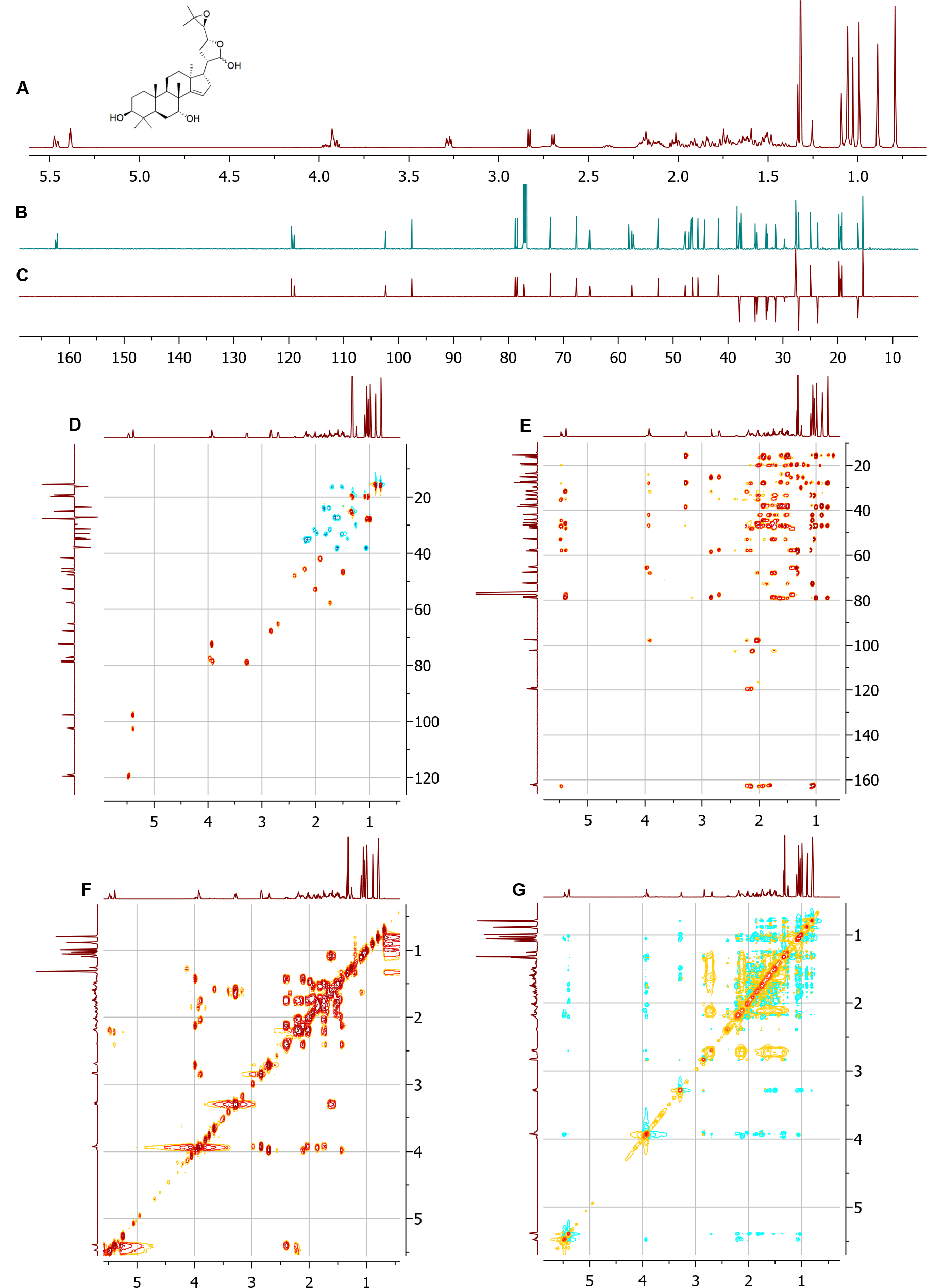


# Figure 1. NMR spectra of apo-melianol (3) (*M. azedarach*).

NMR spectra of C21 epimeric mixture ([CDCl3], δ (ppm)). (A) 1H. (B) 13C. (C) DEPT-135. (D) DEPT-edited-HSQC. (E) HMBC. (F) COSY. (G) NOESY*.*


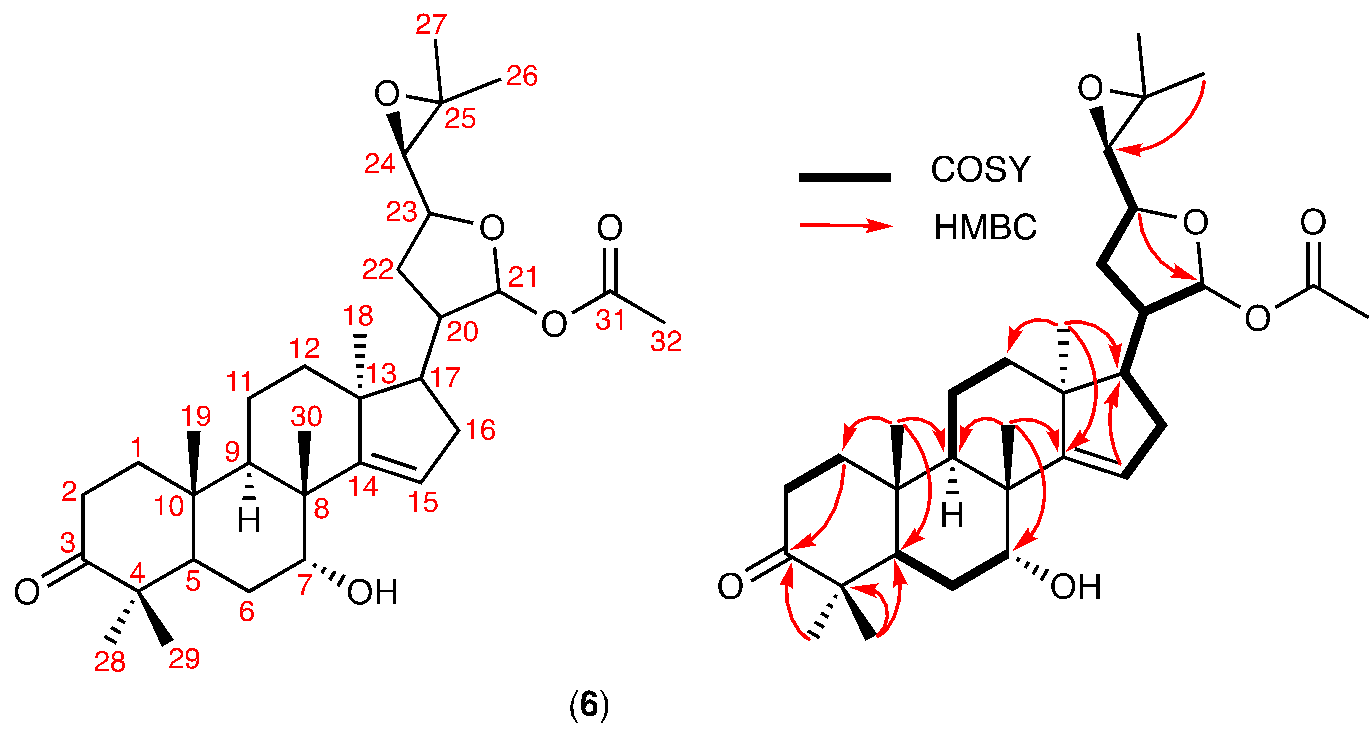


# Figure 2. NMR Assignment of (6) (*C. sinensis*).

Assignment based on NMR spectra ([CDCl_3_], δ (ppm)) listed in Figure 3-7.


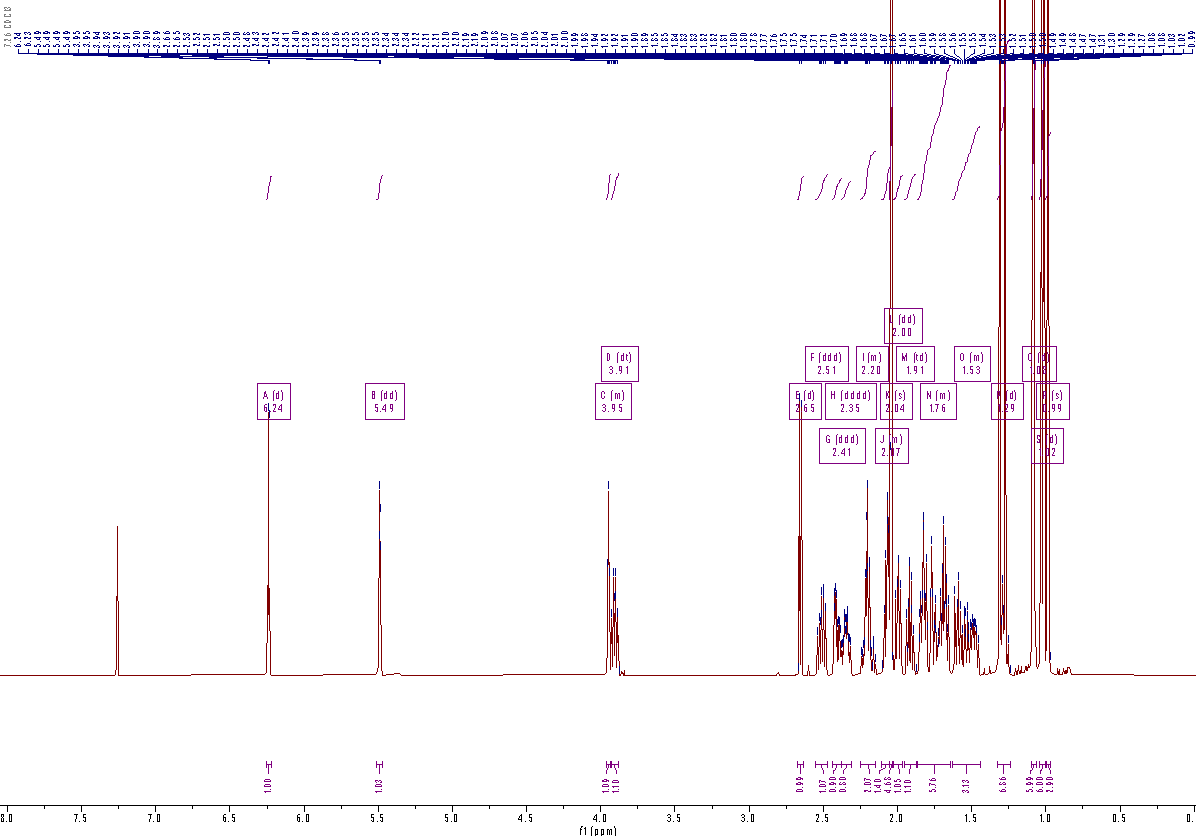


# Figure 3. ^1^H spectrum for (6) (*C. sinensis*).

.


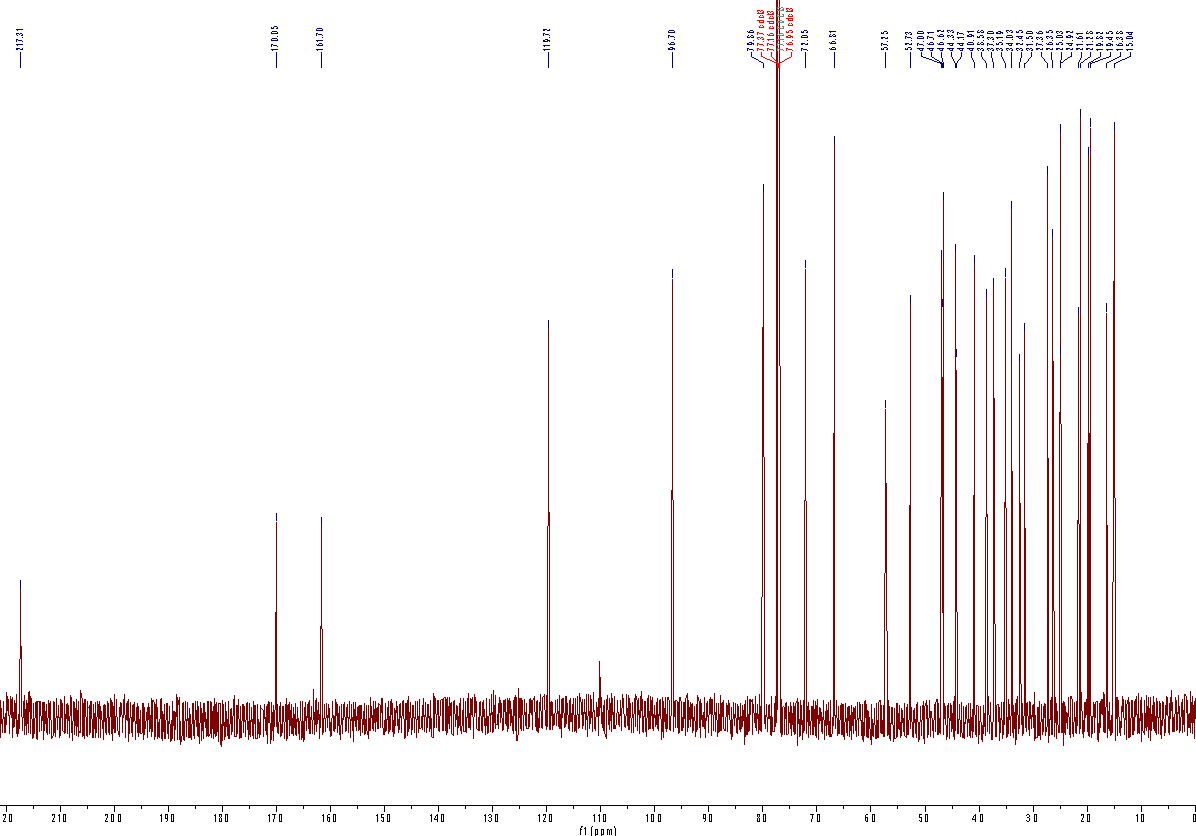


# Figure 4. ^13^C spectrum for (6) (*C. sinensis*).

.


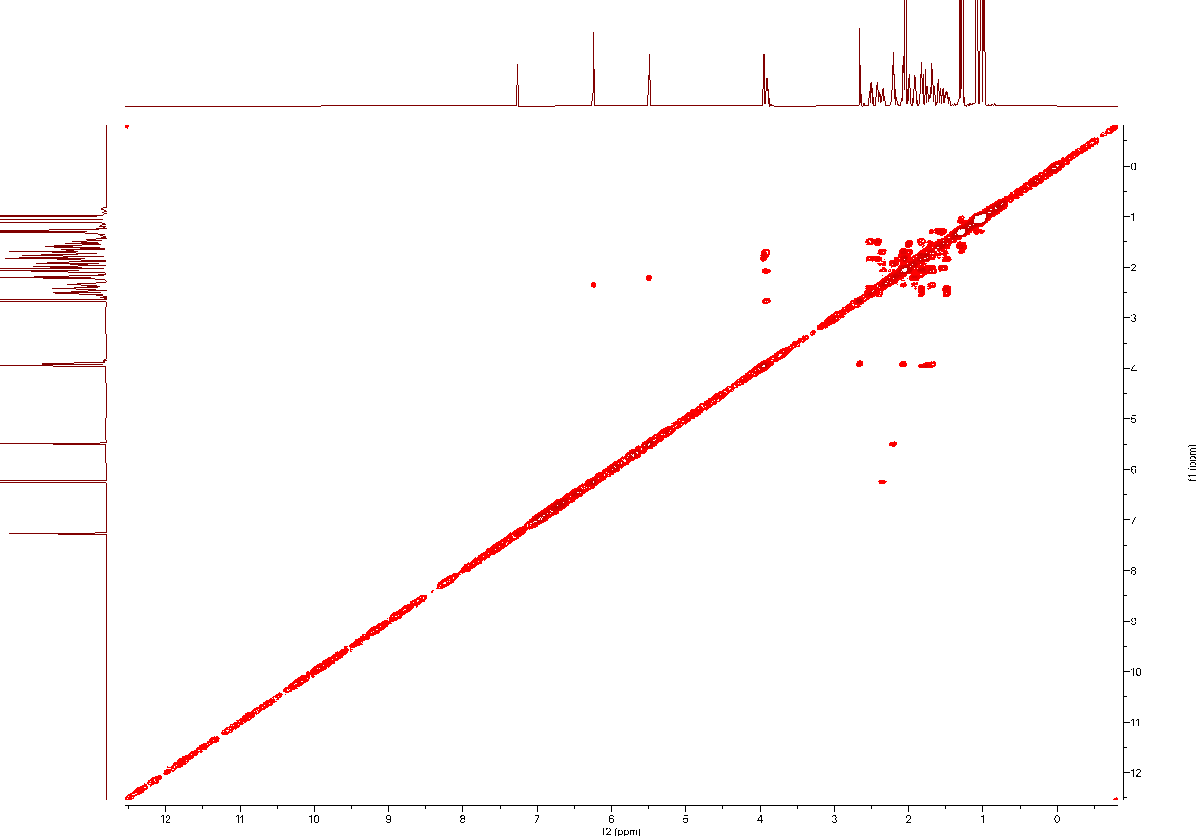


# Figure 5. COSY spectrum for (6) (*C. sinensis*).

.


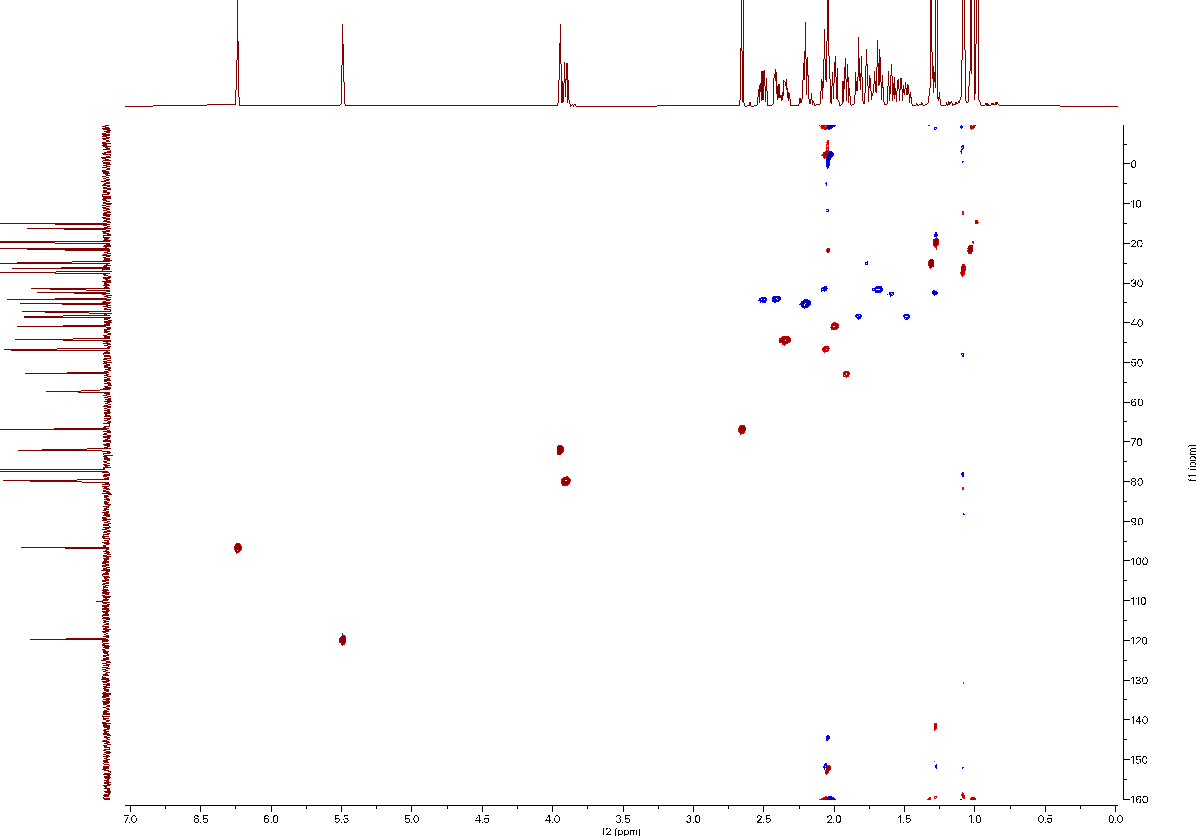


# Figure 6. HSQC spectrum for (6) (*C. sinensis*).

.


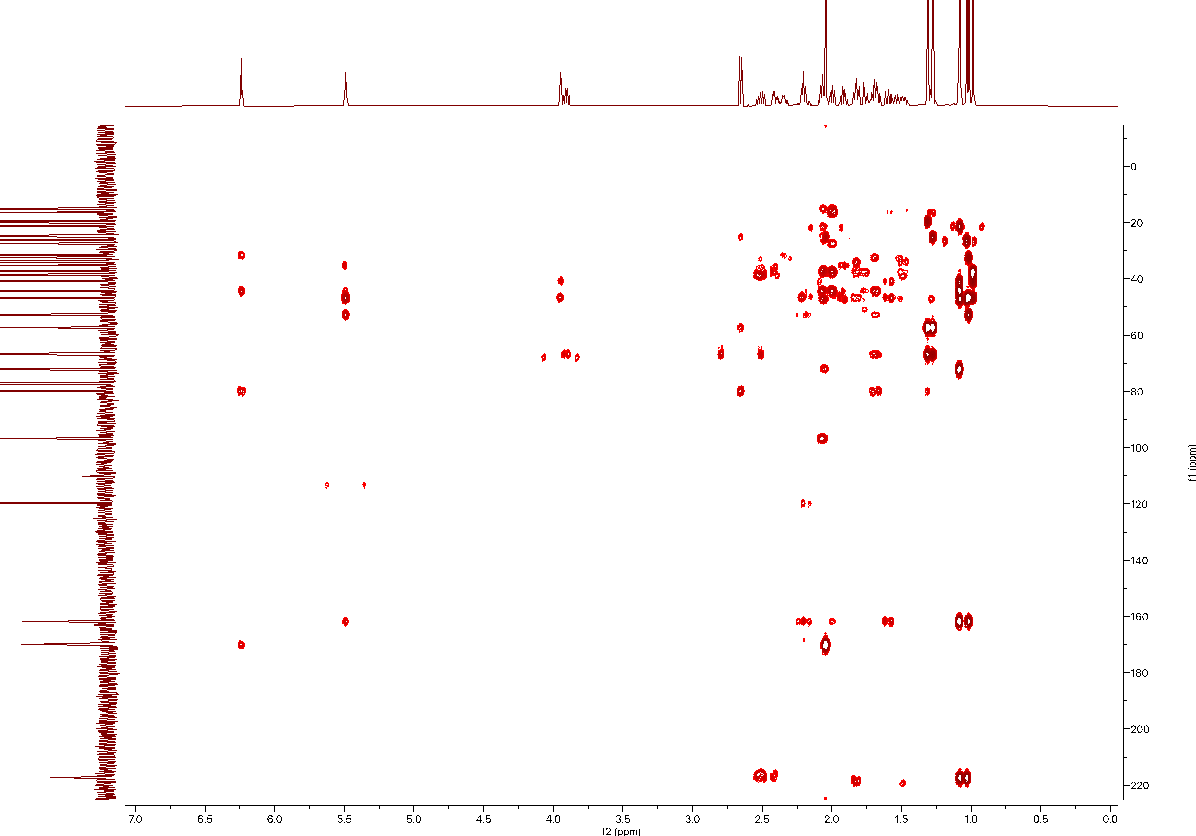


# Figure 7. HMBC spectrum for (6) (*C. sinensis*).


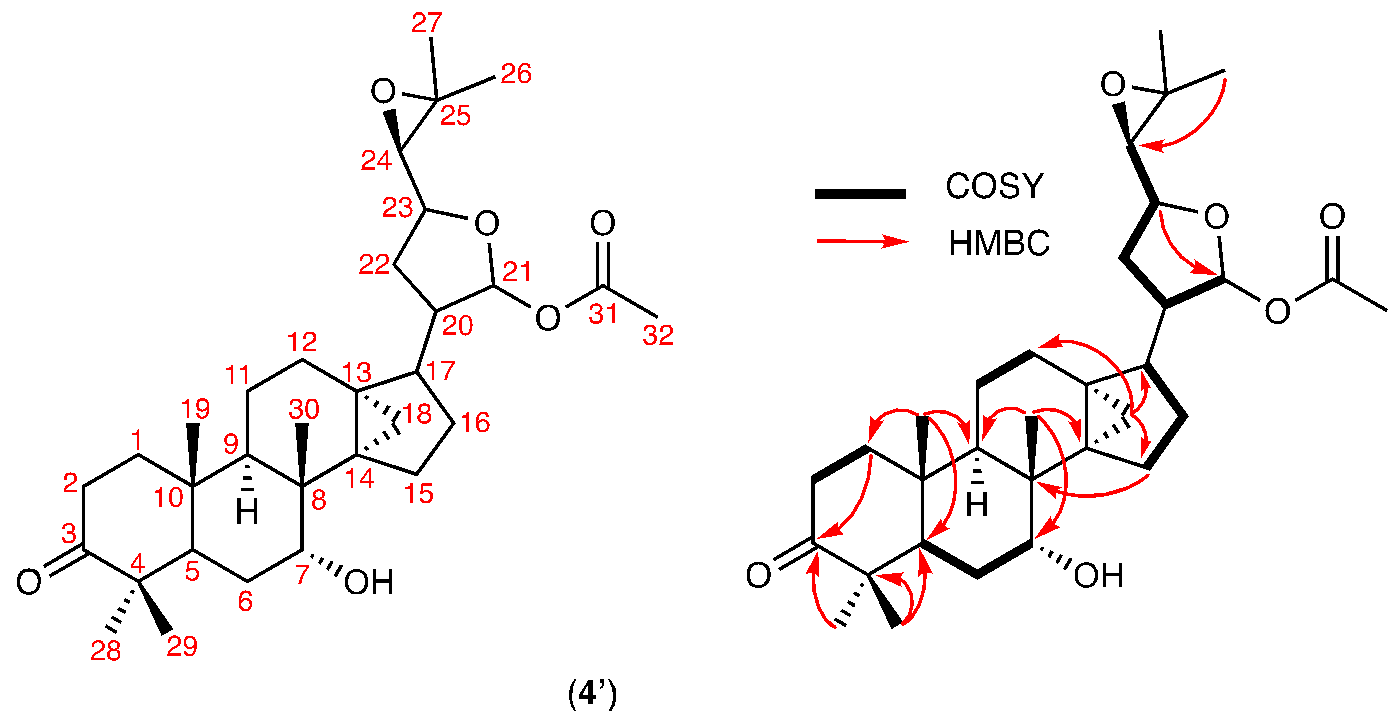


# Figure 8. NMR Assignment of (4’) (*C. sinensis*).

Assignment based on NMR spectra ([CDCl_3_], δ (ppm)) listed in Figure 9-13.


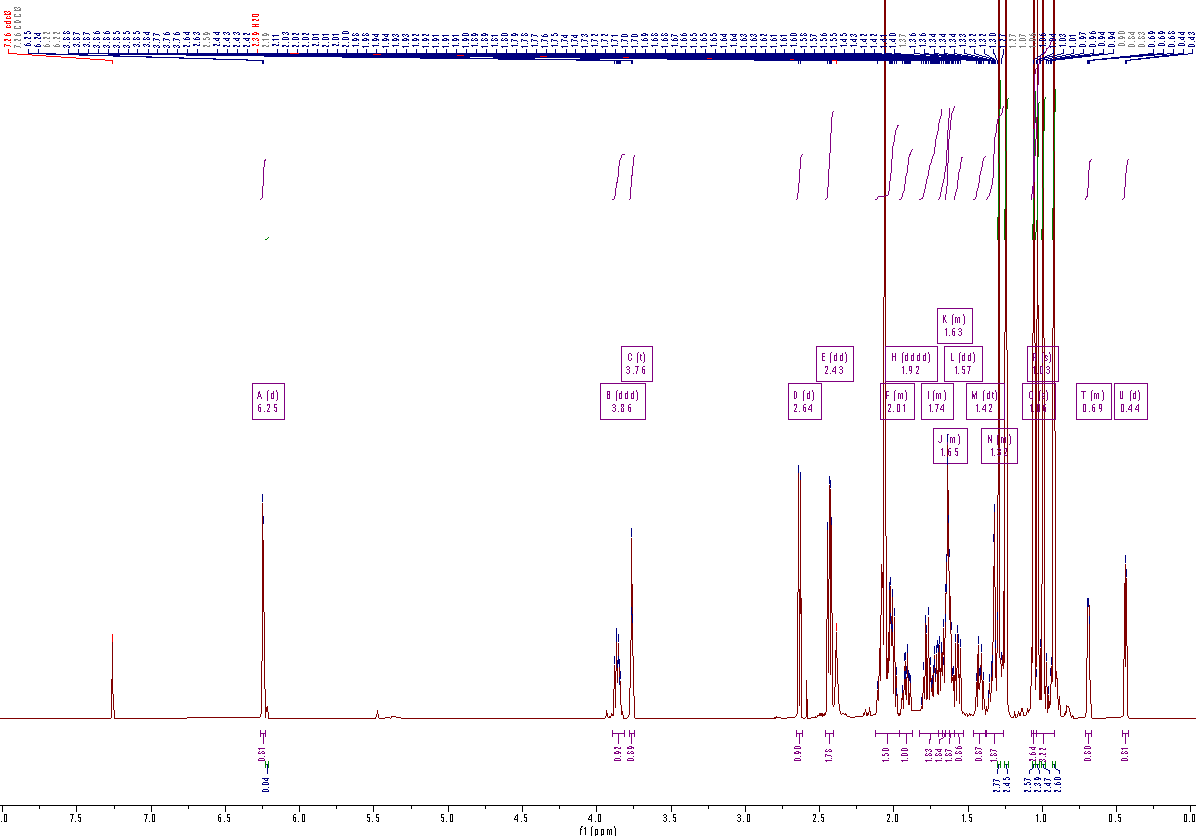


# Figure 9. ^1^H spectrum for (4’) (*C. sinensis*).


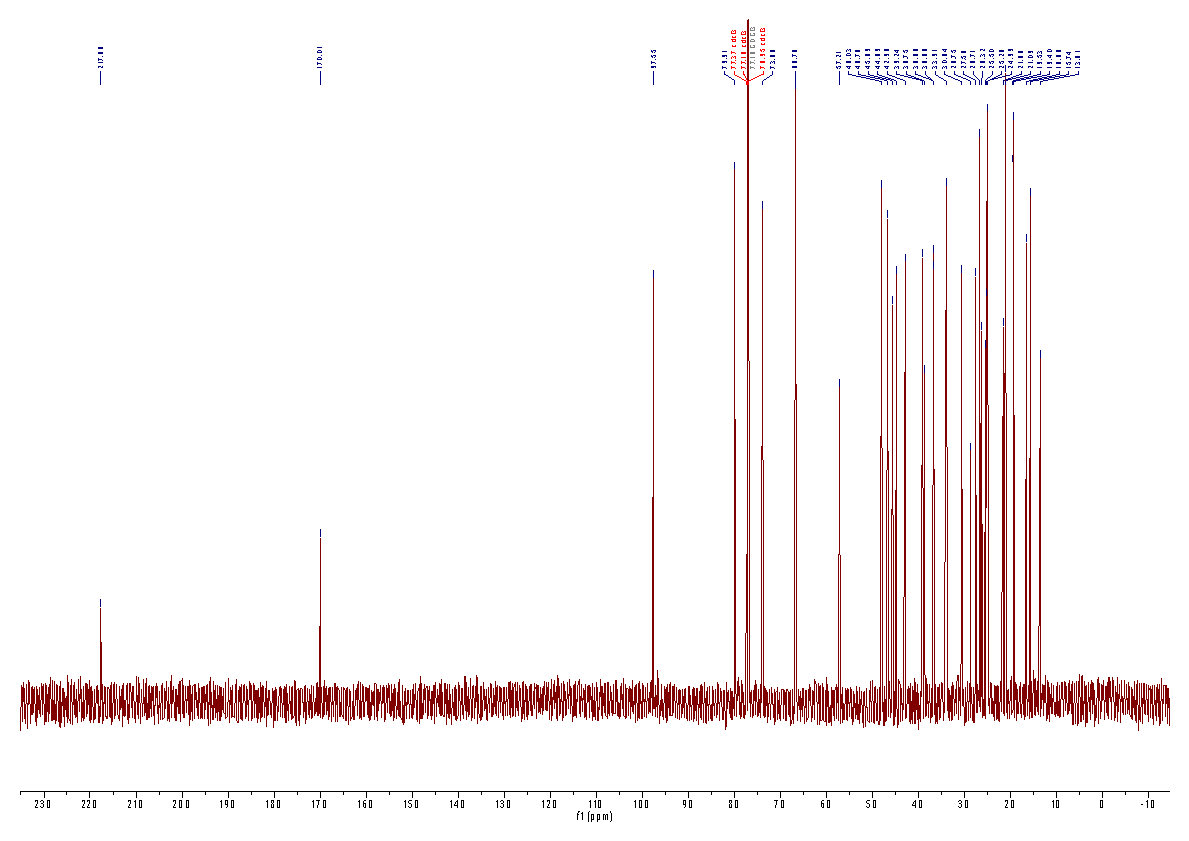


# Figure 10. ^13^C spectrum for (4’) (*C. sinensis*).


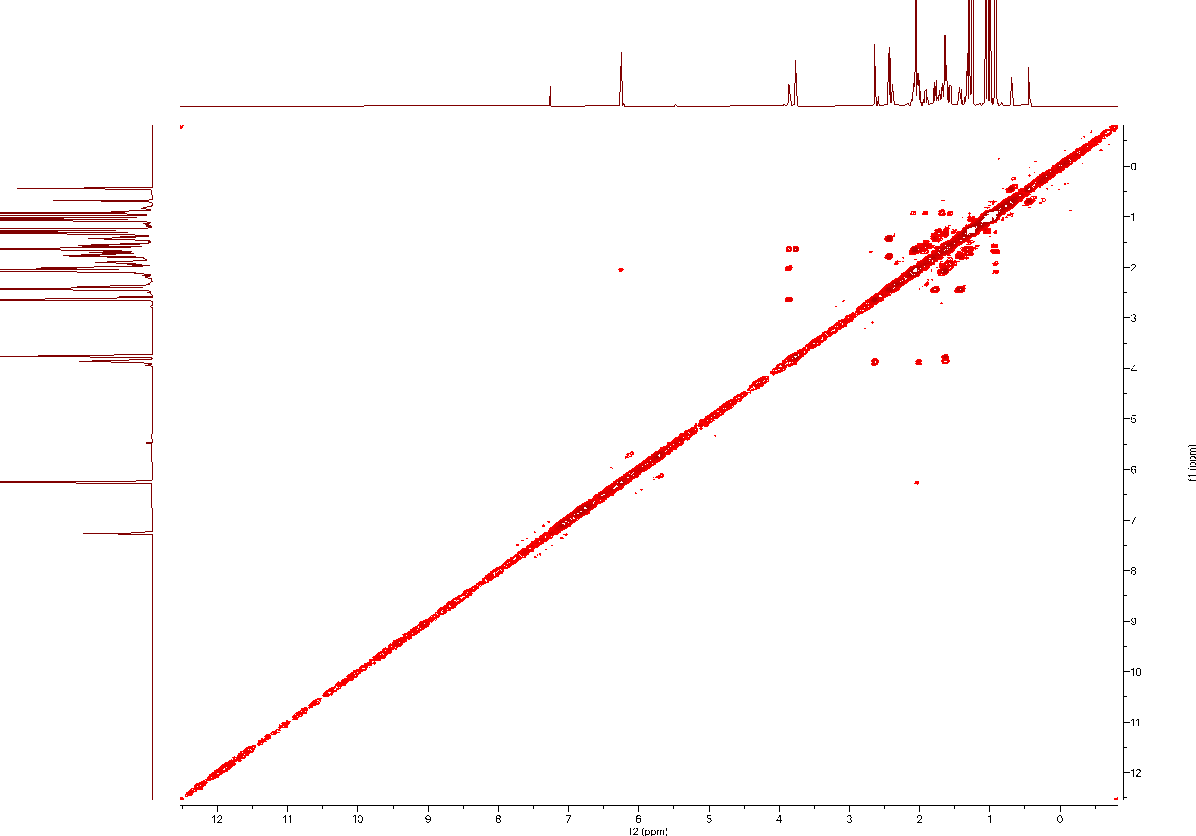


# Figure 11. COSY spectrum for (4’) (*C. sinensis*).


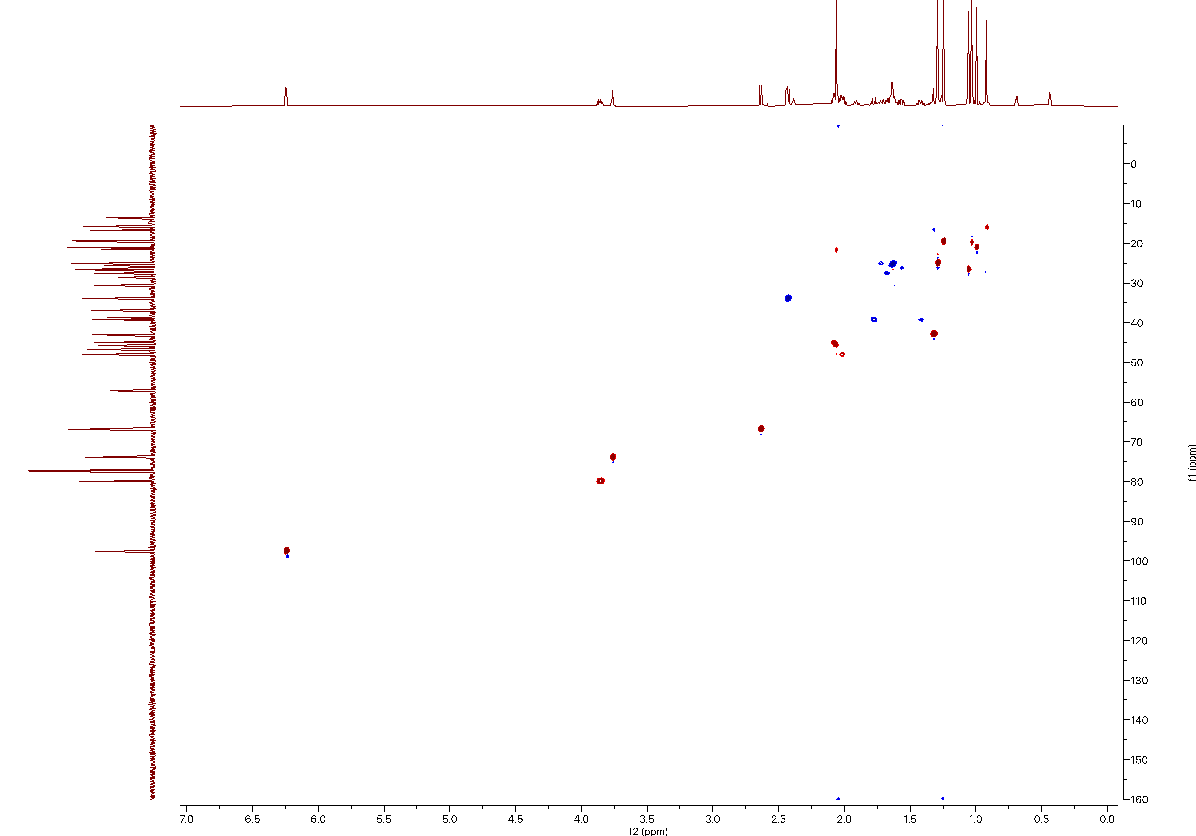


# Figure 12. HSQC spectrum for (4’) (*C. sinensis*).


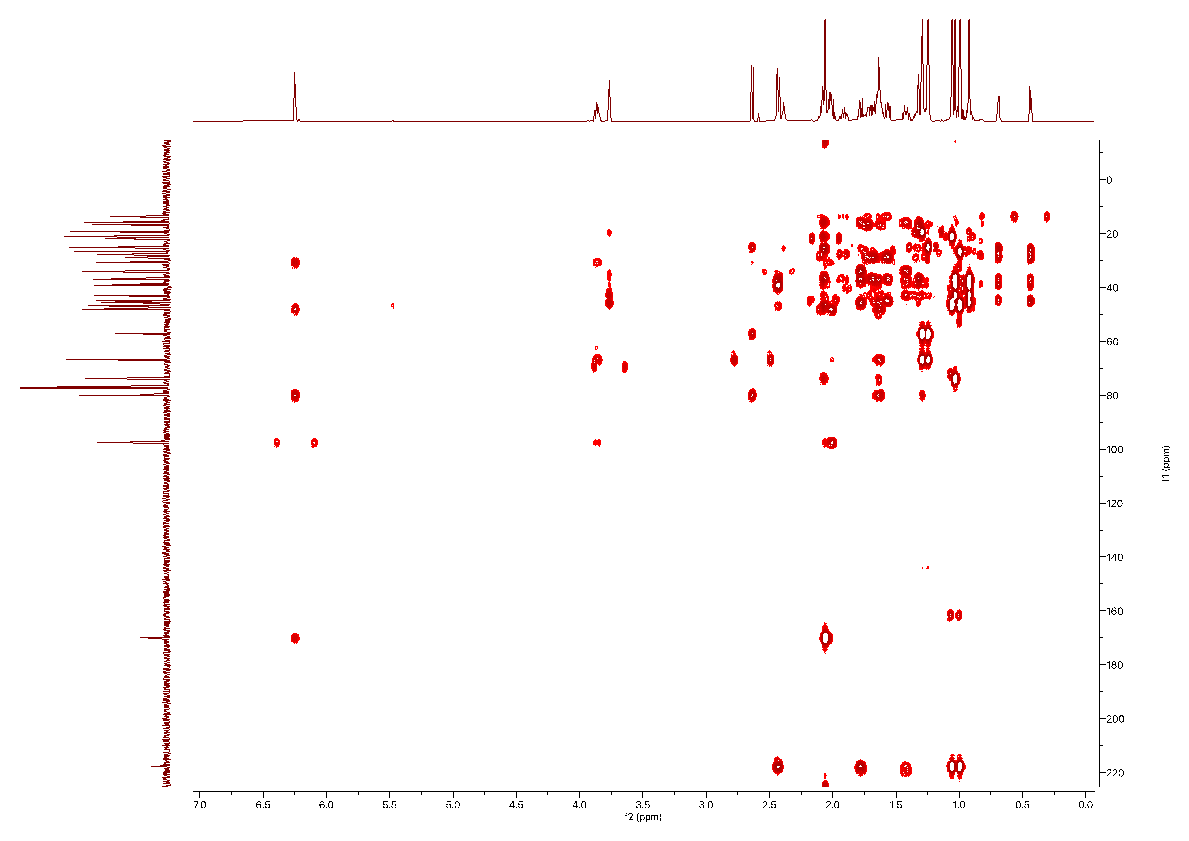


# Figure 13. HMBC spectrum for (4’) (*C. sinensis*).


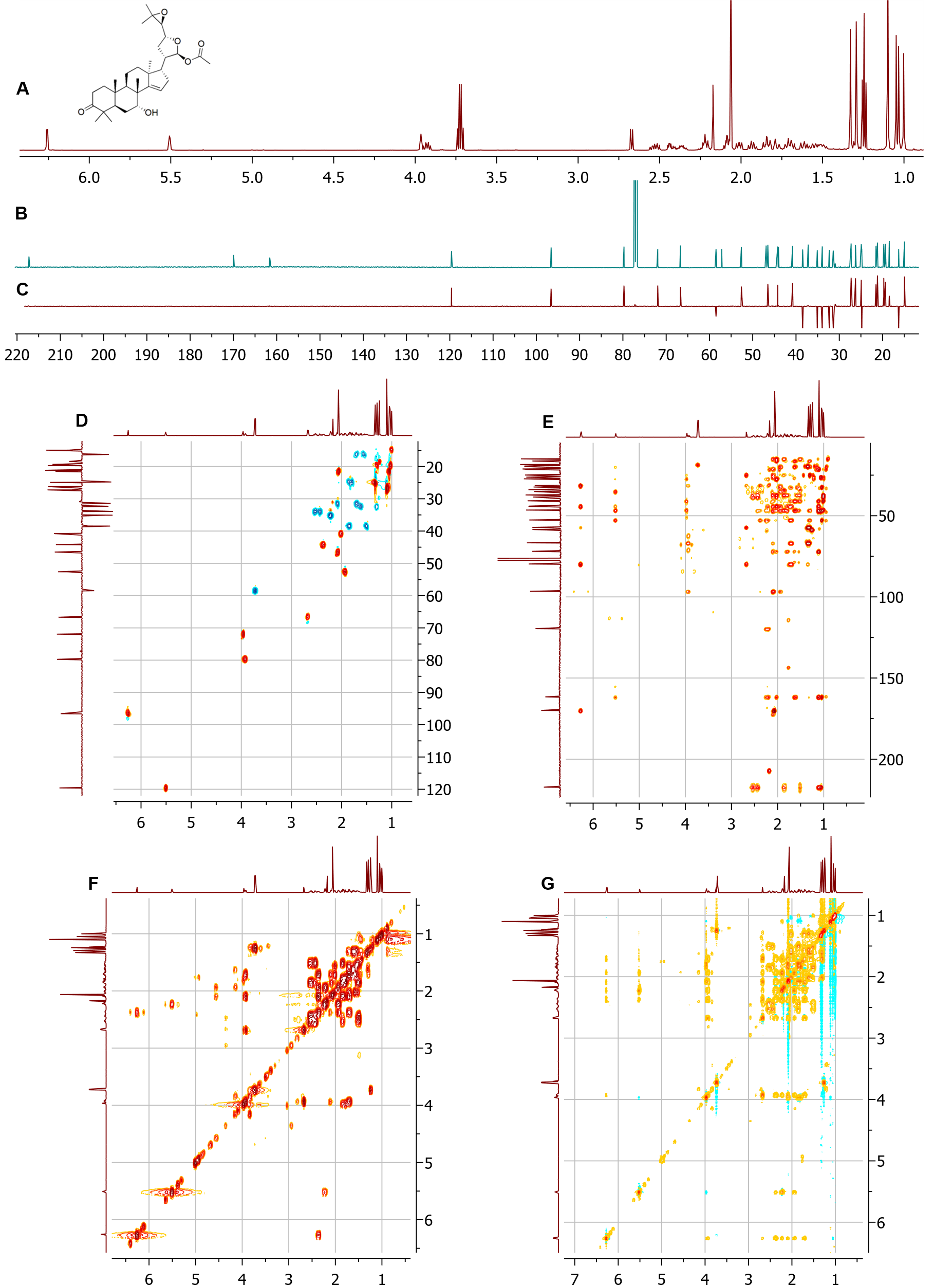


# Figure 14. NMR spectra of 21(*S*)-acetoxyl-apo-melianone (6) (*M. azedarach*).

NMR spectra **(**[CDCl_3_], δ (ppm)).(A) ^1^H. (B) ^13^C. (C) DEPT-135. (D) DEPT-edited-HSQC. (E) HMBC. (F) COSY. (G) NOESY.


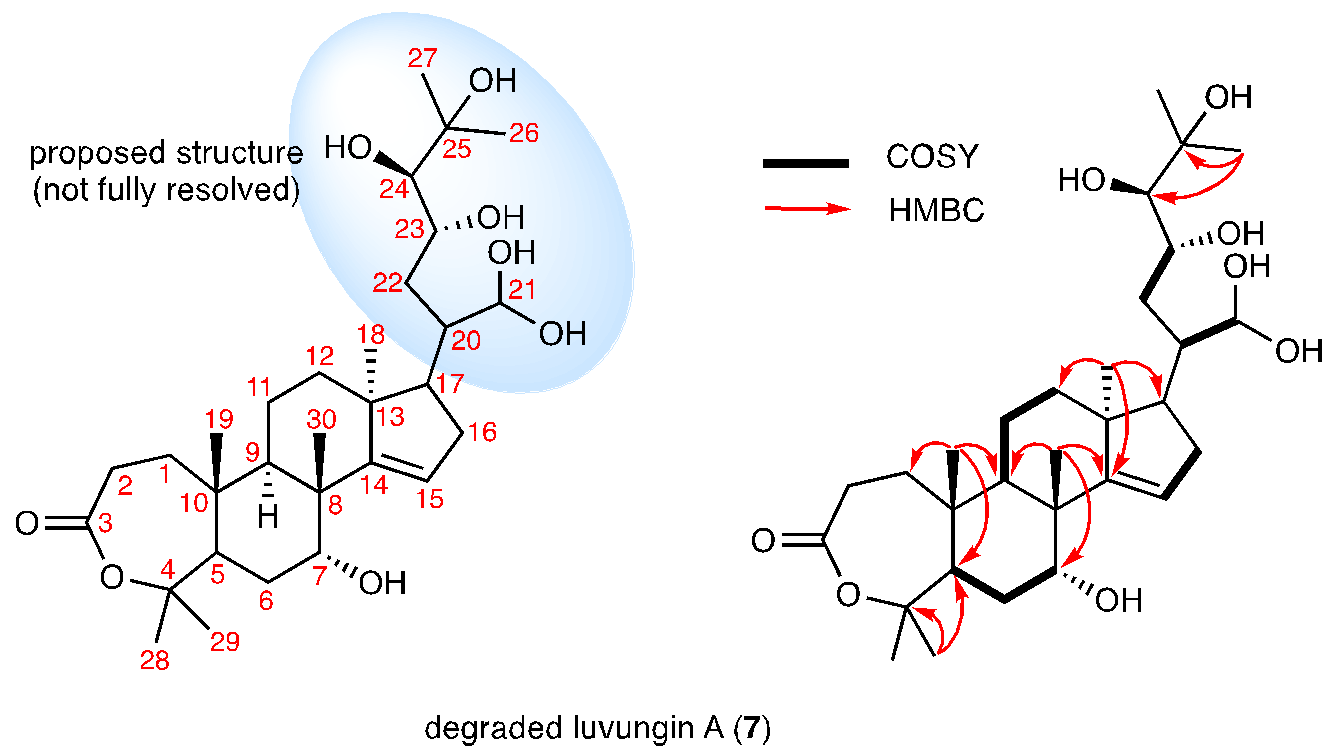


# Figure 15. NMR assignment of degraded luvungin A (7) (*C. sinensis*).

Assignment based on NMR spectra ([CDCl_3_], δ (ppm)) listed in Figure 16-20.

**
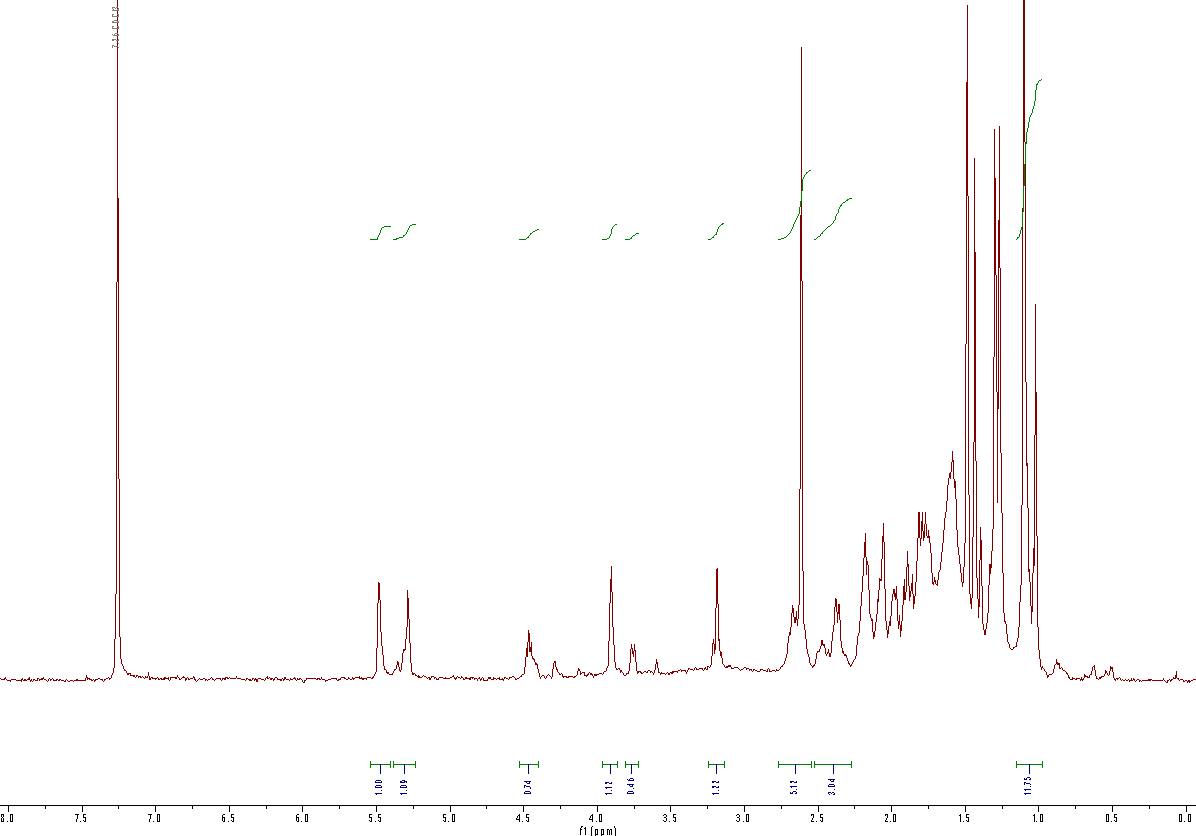
**

# Figure 16. ^1^H spectrum for degraded (7) (*C. sinensis*).


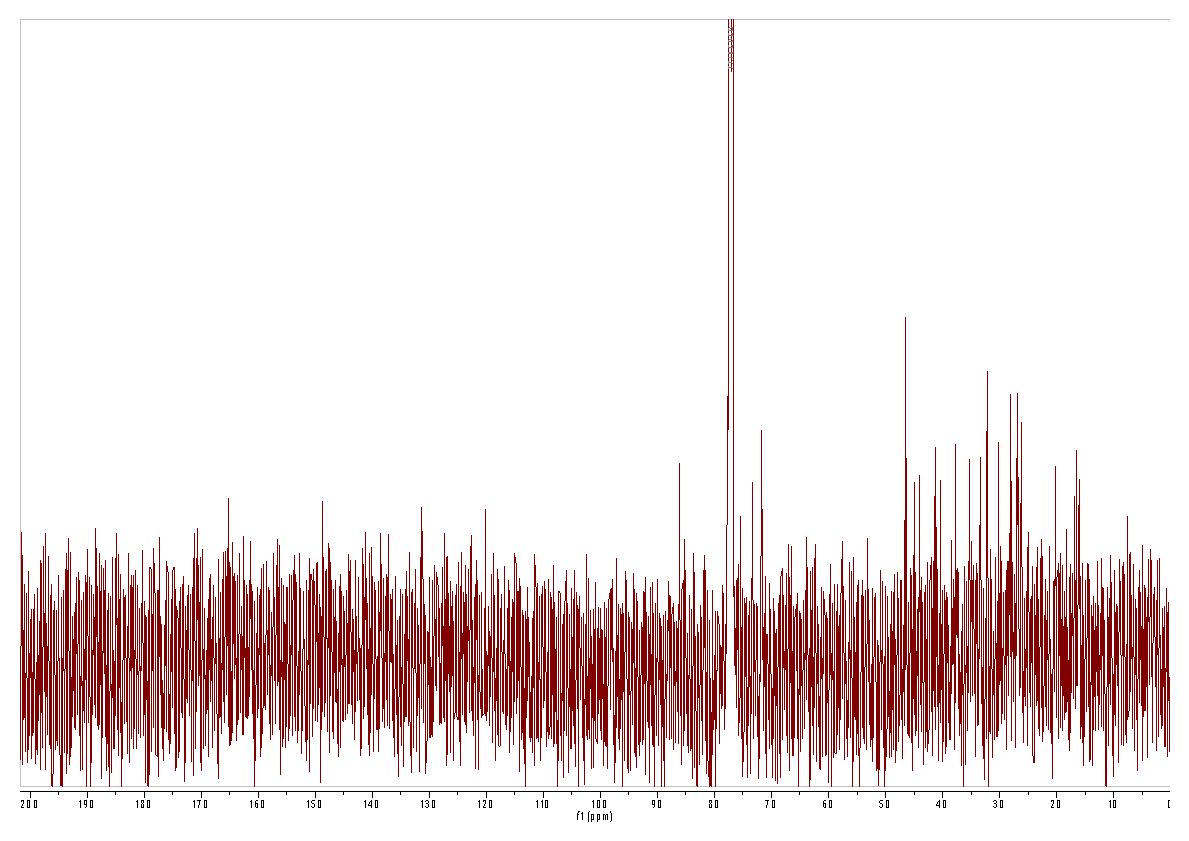


# Figure 17. ^13^C spectrum for degraded (7) (*C. sinensis*).


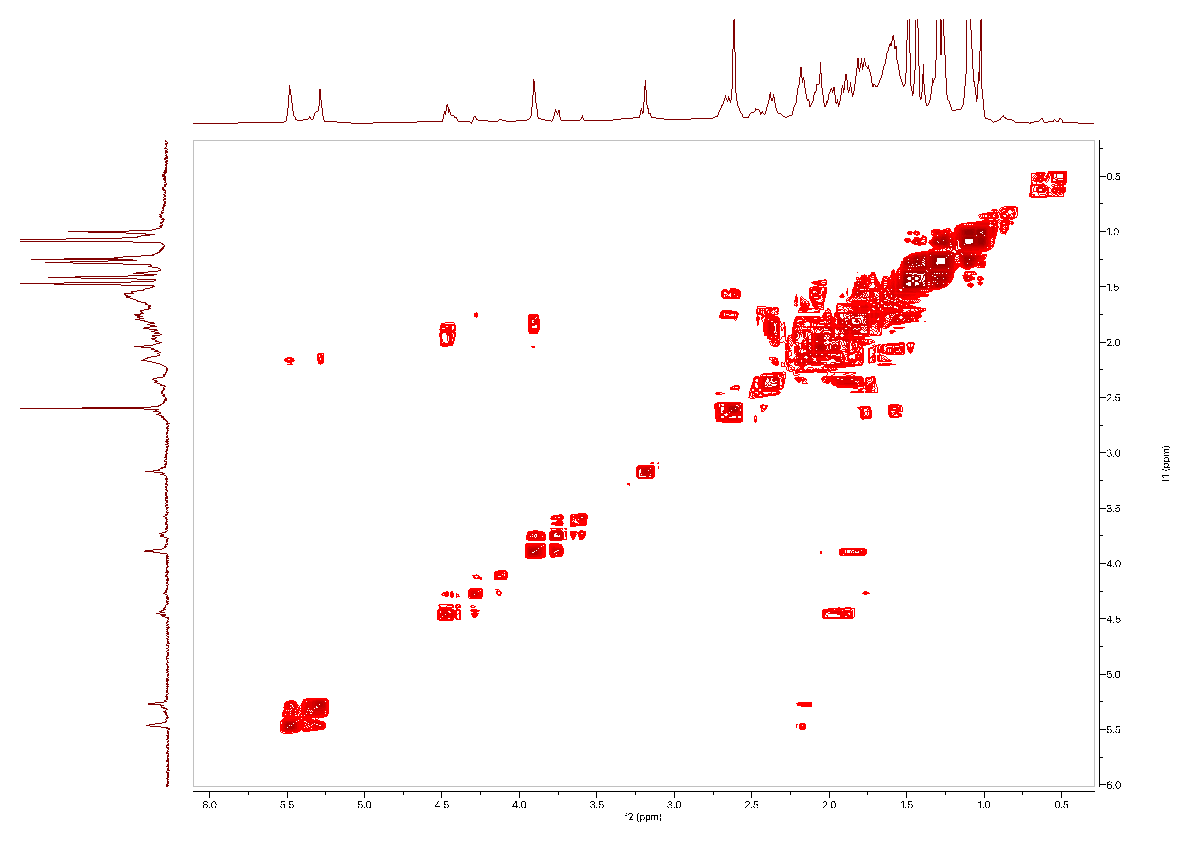


# Figure 18. COSY spectrum for degraded (7) (*C. sinensis*).


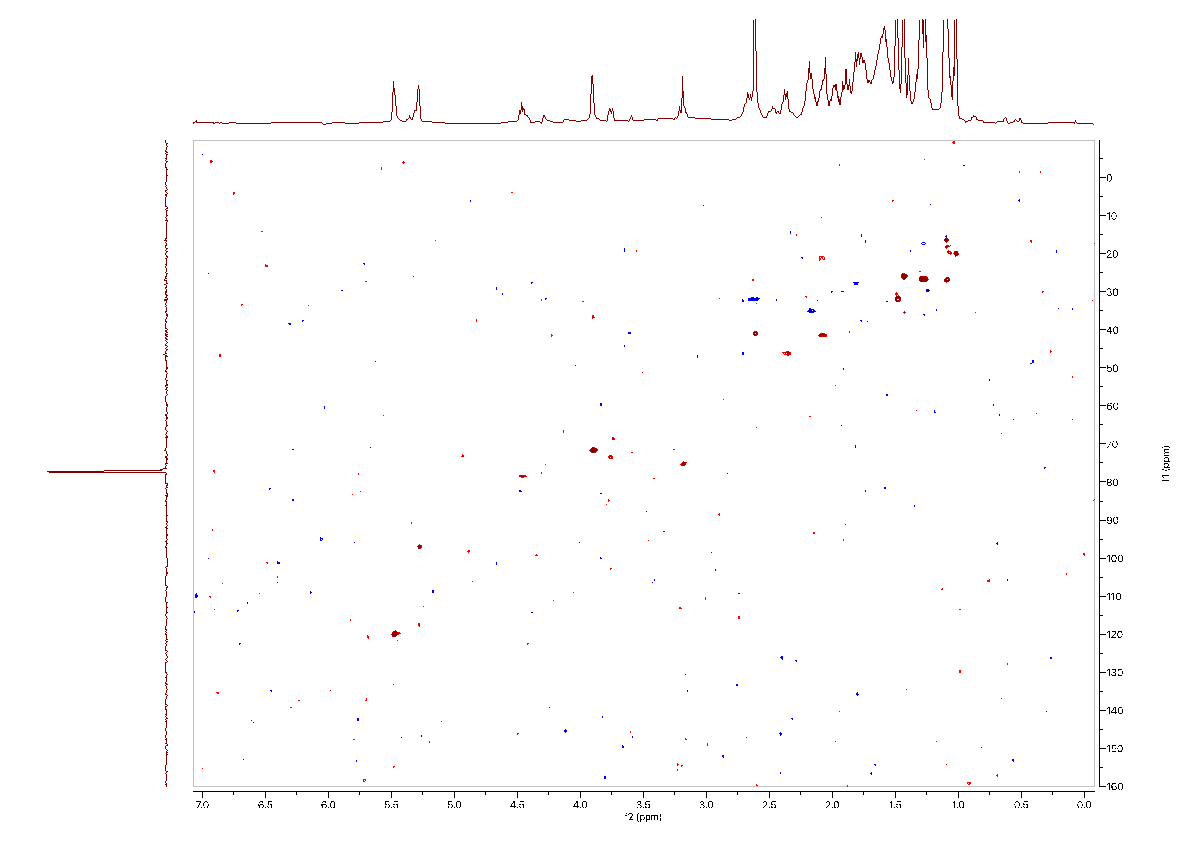


# Figure 19. HSQC spectrum for degraded (7) (*C. sinensis*).


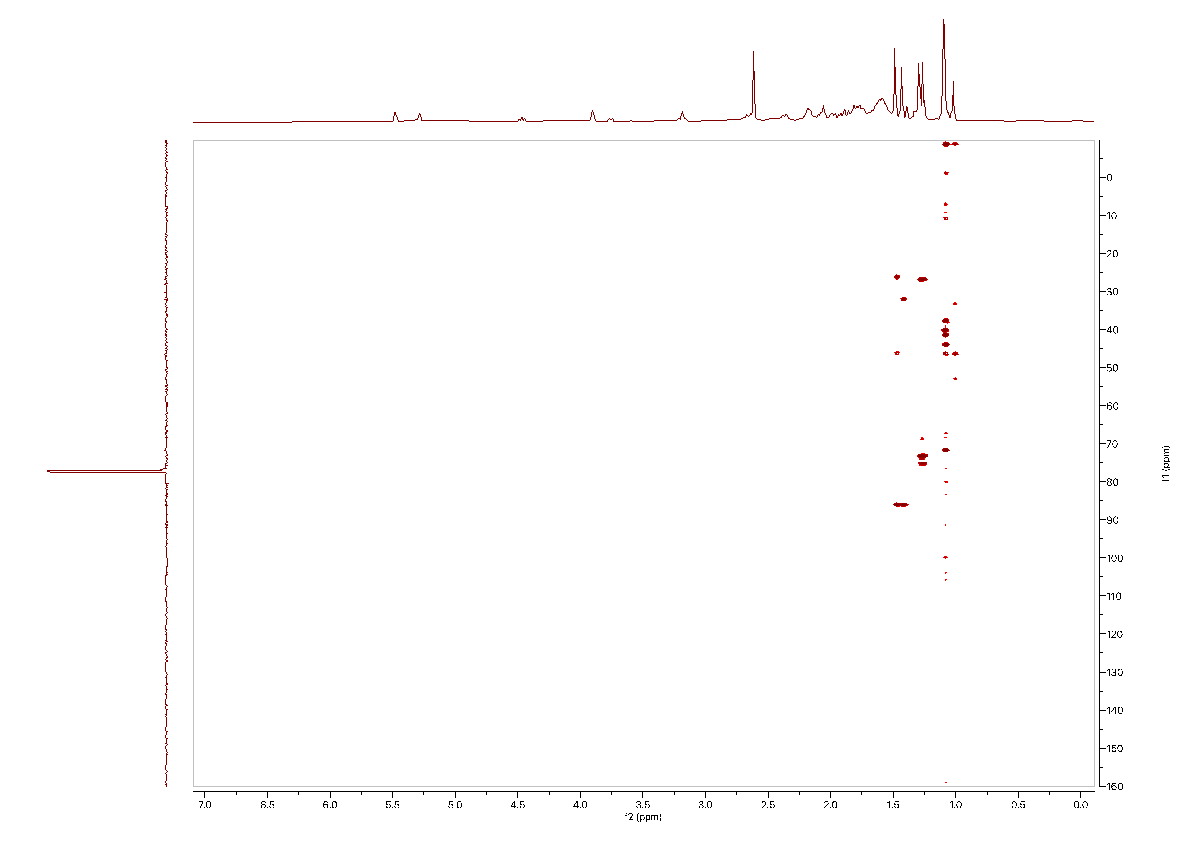


# Figure 20. HMBC spectrum for degraded (7) (*C. sinensis*).


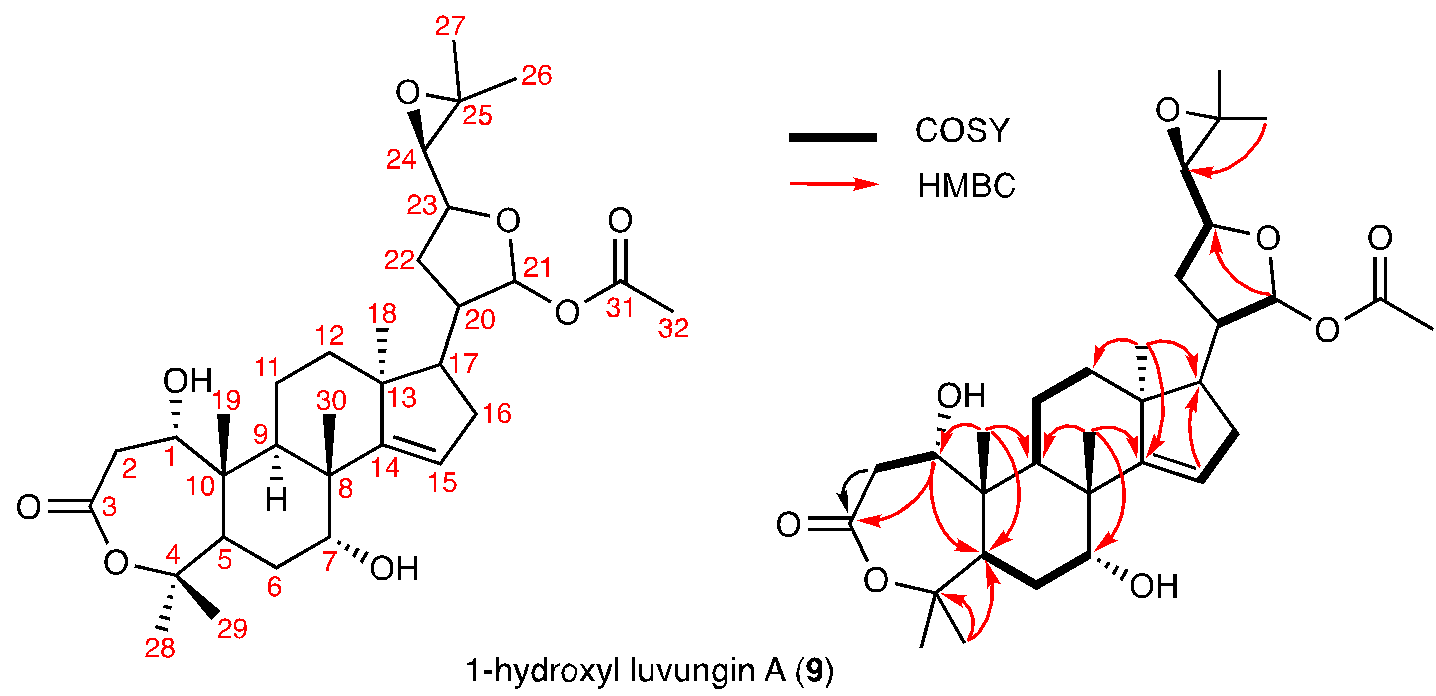


# Figure 21. NMR assignment of 1-hydroxyl luvungin A (9) (*C. sinensis*).

Assignment based on NMR spectra ([CDCl_3_], δ (ppm)) listed in Figure 22-26.


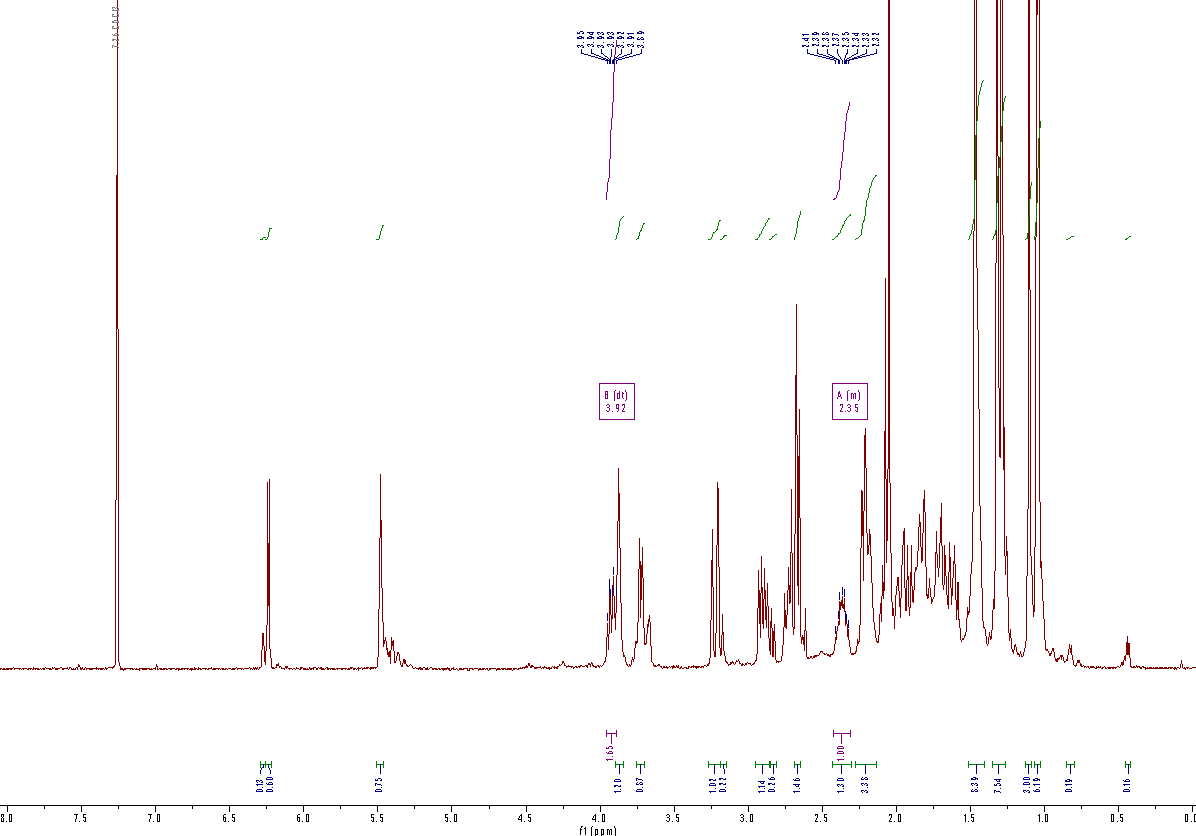


# Figure 22. ^1^H spectrum for (9) (*C. sinensis*).


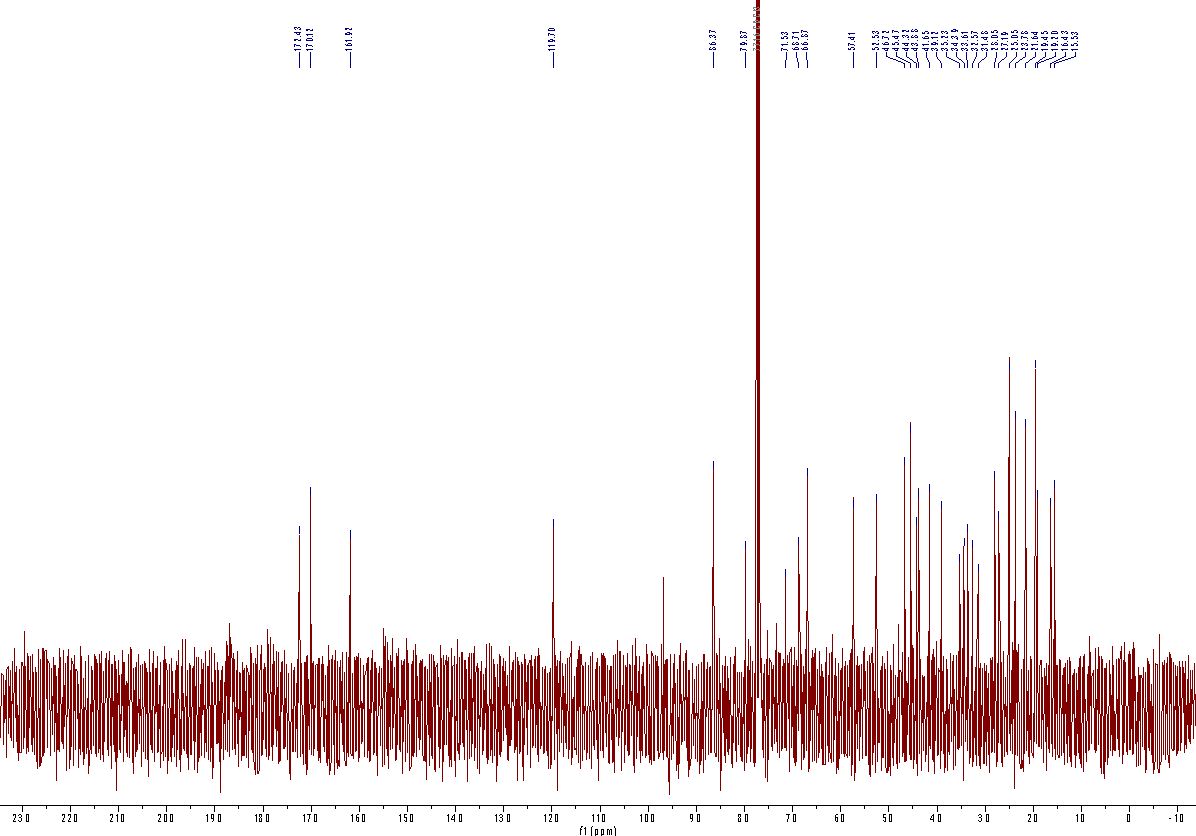


# Figure 23. ^13^C spectrum for (9) (*C. sinensis*).


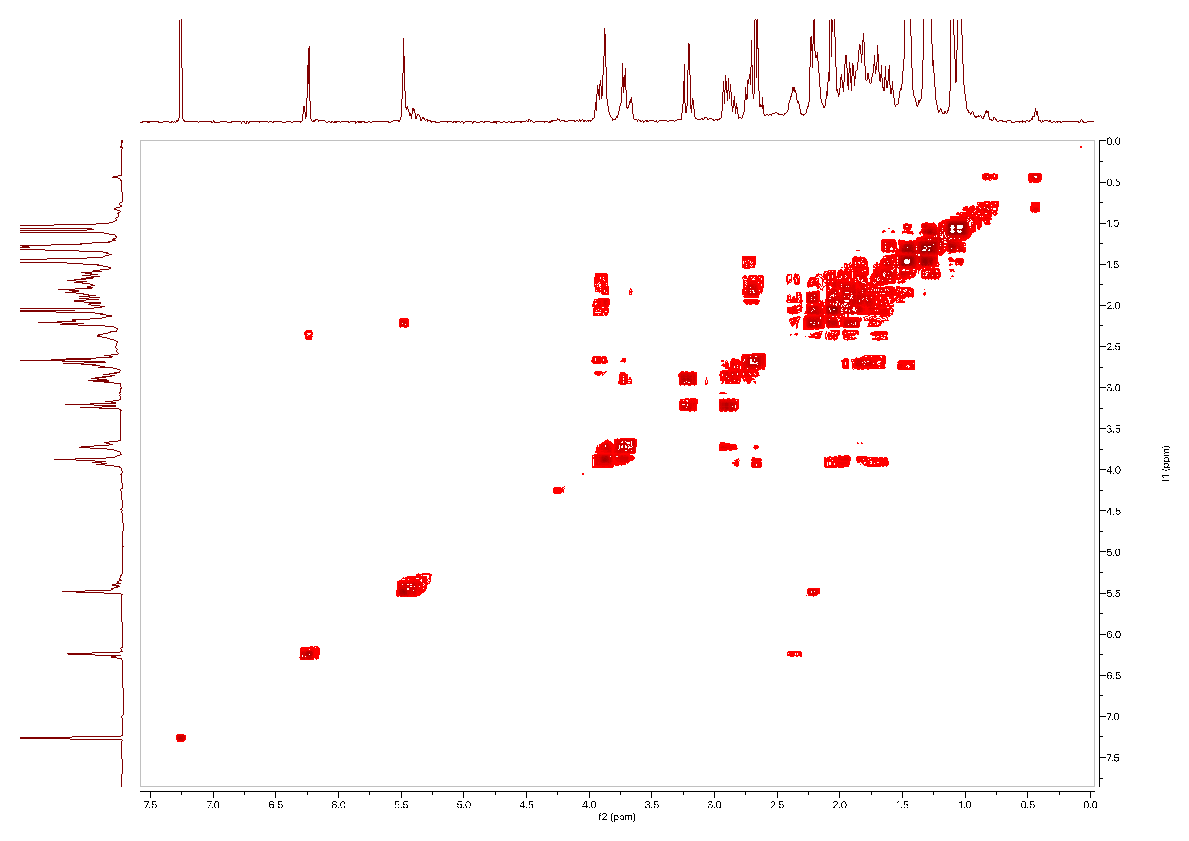


# Figure 24. COSY spectrum for (9) (*C. sinensis*).


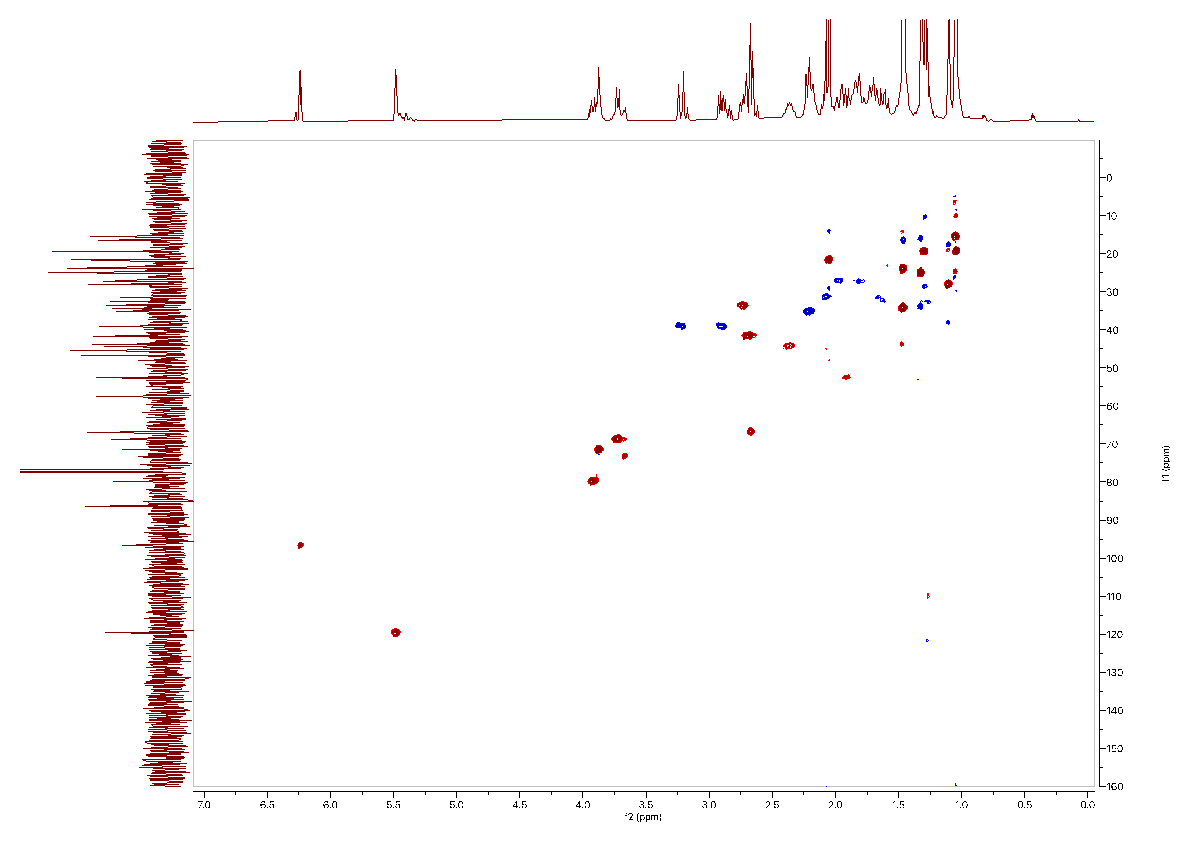


# Figure 25. HSQC spectrum for (9) (*C. sinensis*).


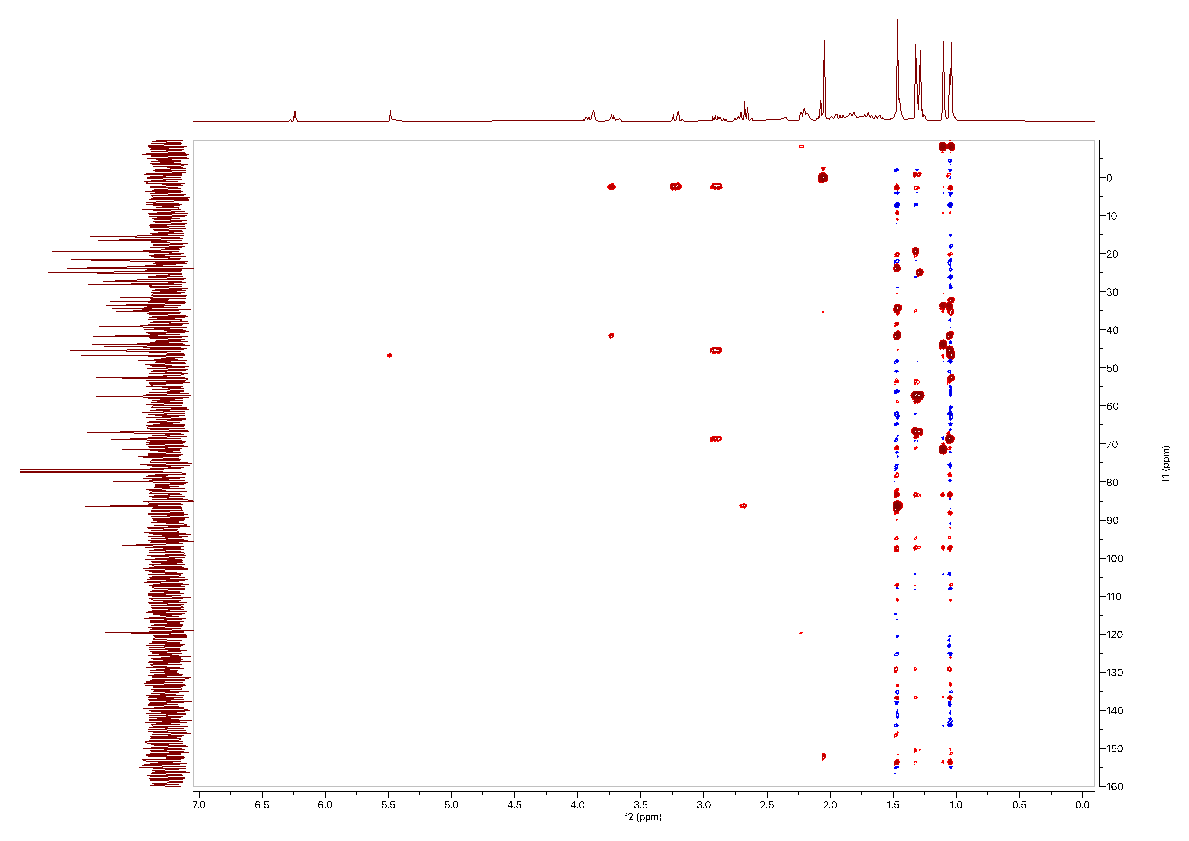


# Figure 26. HMBC spectrum for (9) (*C. sinensis*).


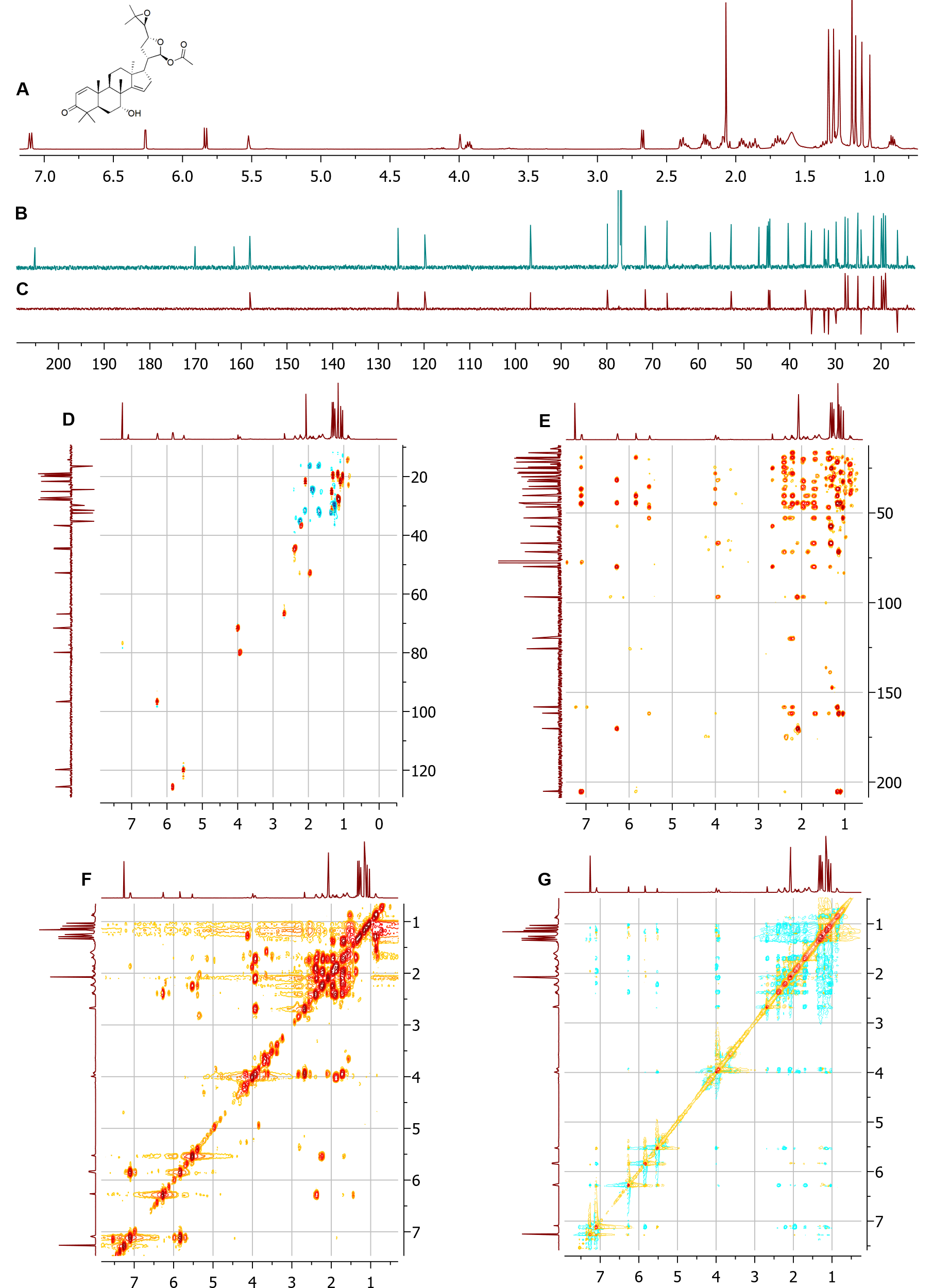


# Figure 27*.* NMR spectra of epi-neemfruitin B (10) *(M. azedarach).*

NMR spectra ([CDCl_3_], δ (ppm)). (A) ^1^H. (B) ^13^C. (C) DEPT-135. (D) DEPT-edited-HSQC. (E) HMBC. (F) COSY. (G) ROESY.

**
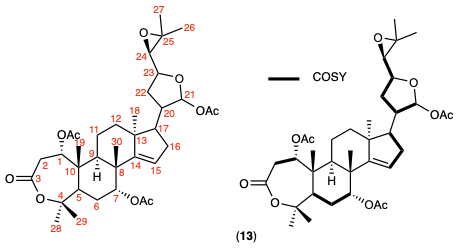
**

# Figure 28. NMR assignment of (13) (*C. sinensis*).

Assignment based on NMR spectra ([CDCl_3_], δ (ppm)) listed in Figure 29-30.


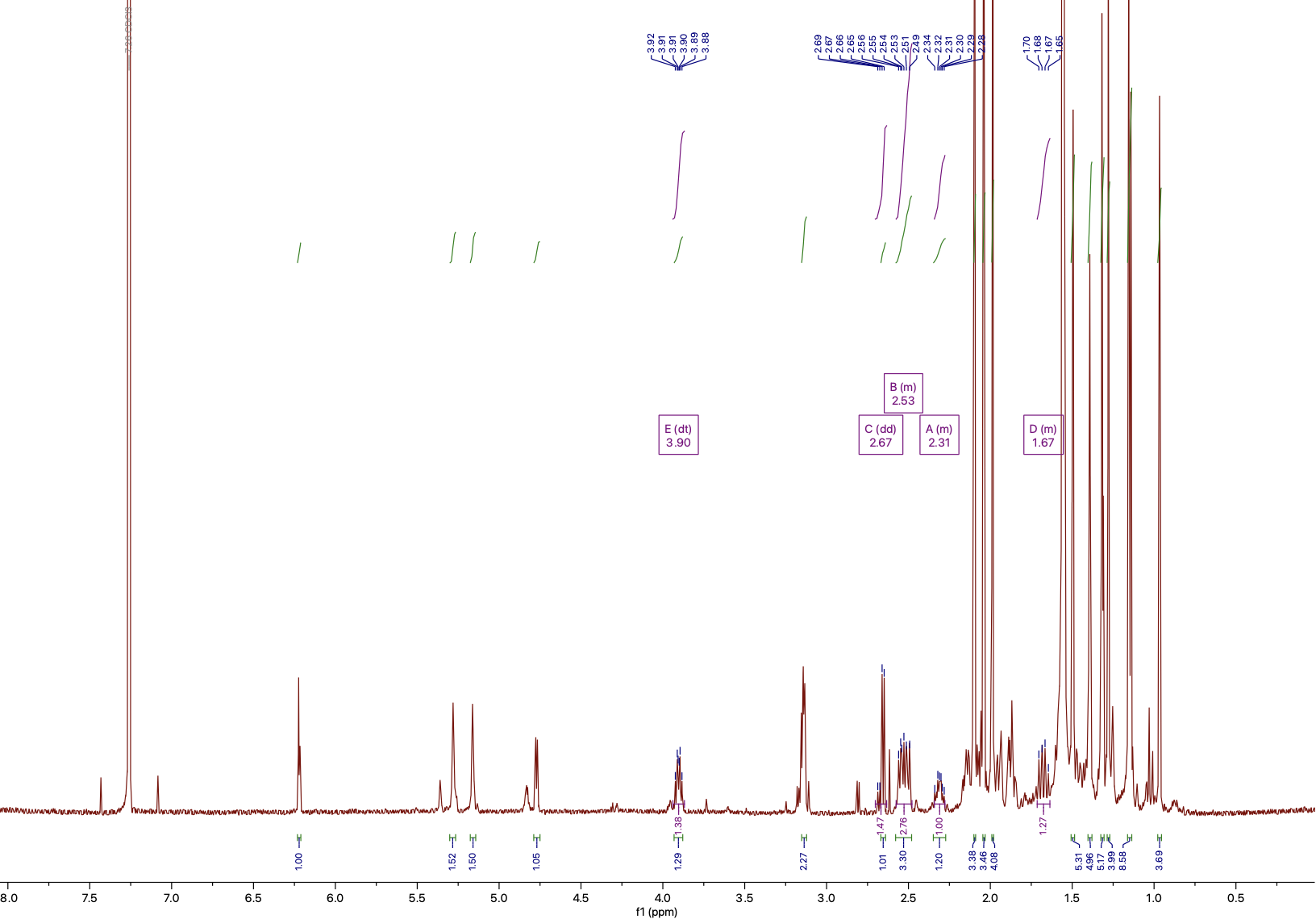


# Figure 29. ^1^H spectrum for (**13**) (*C. sinensis*).


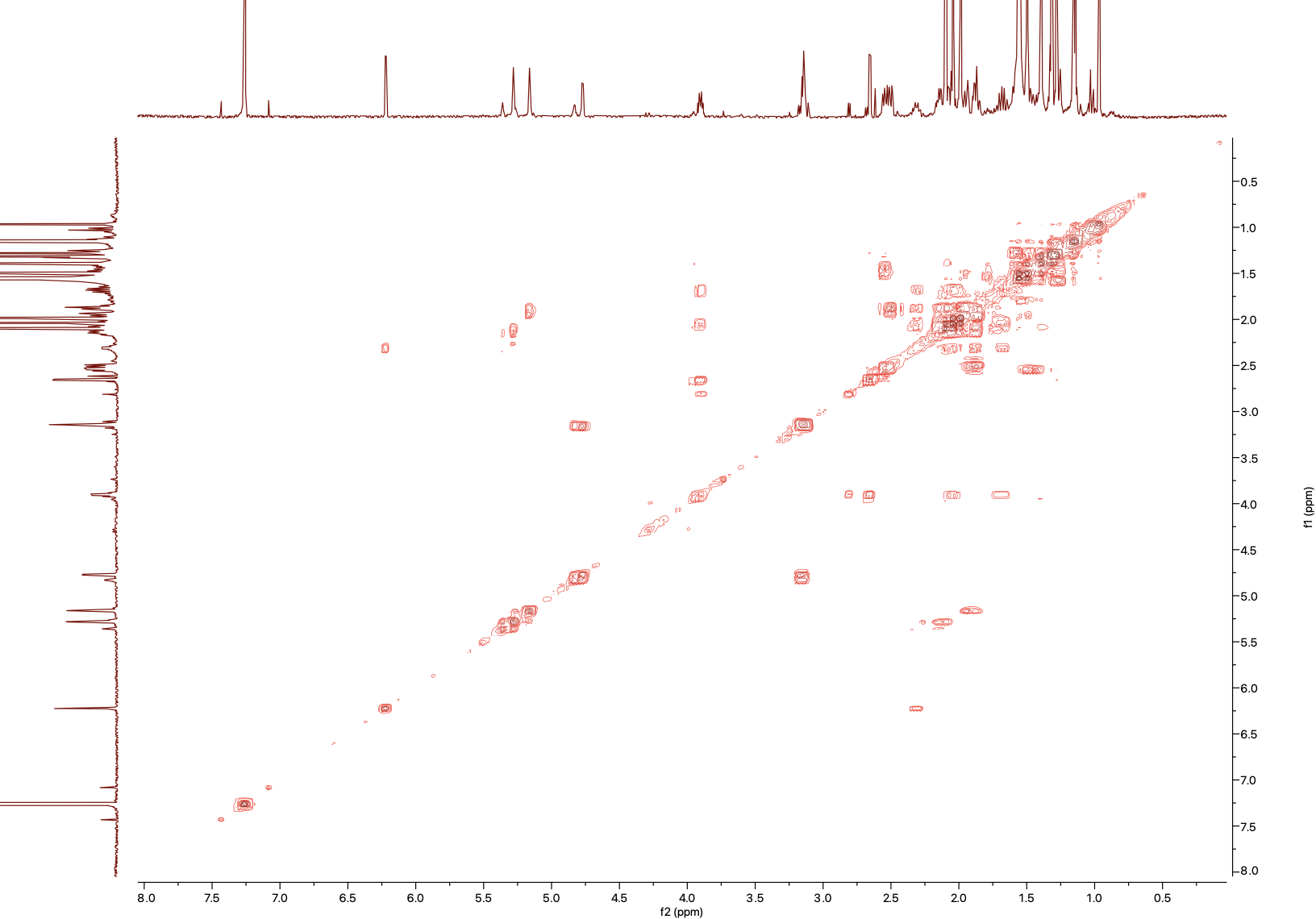


# Figure 30. COSY spectrum of (13) (*C. sinensis*).

#
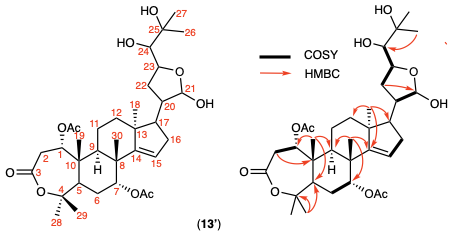


# Figure 31. NMR assignment of (**13’**) (*C. sinensis*).

Assignment based on NMR spectra ([CDCl_3_], δ (ppm)) listed in Figure 32-36.

#

#
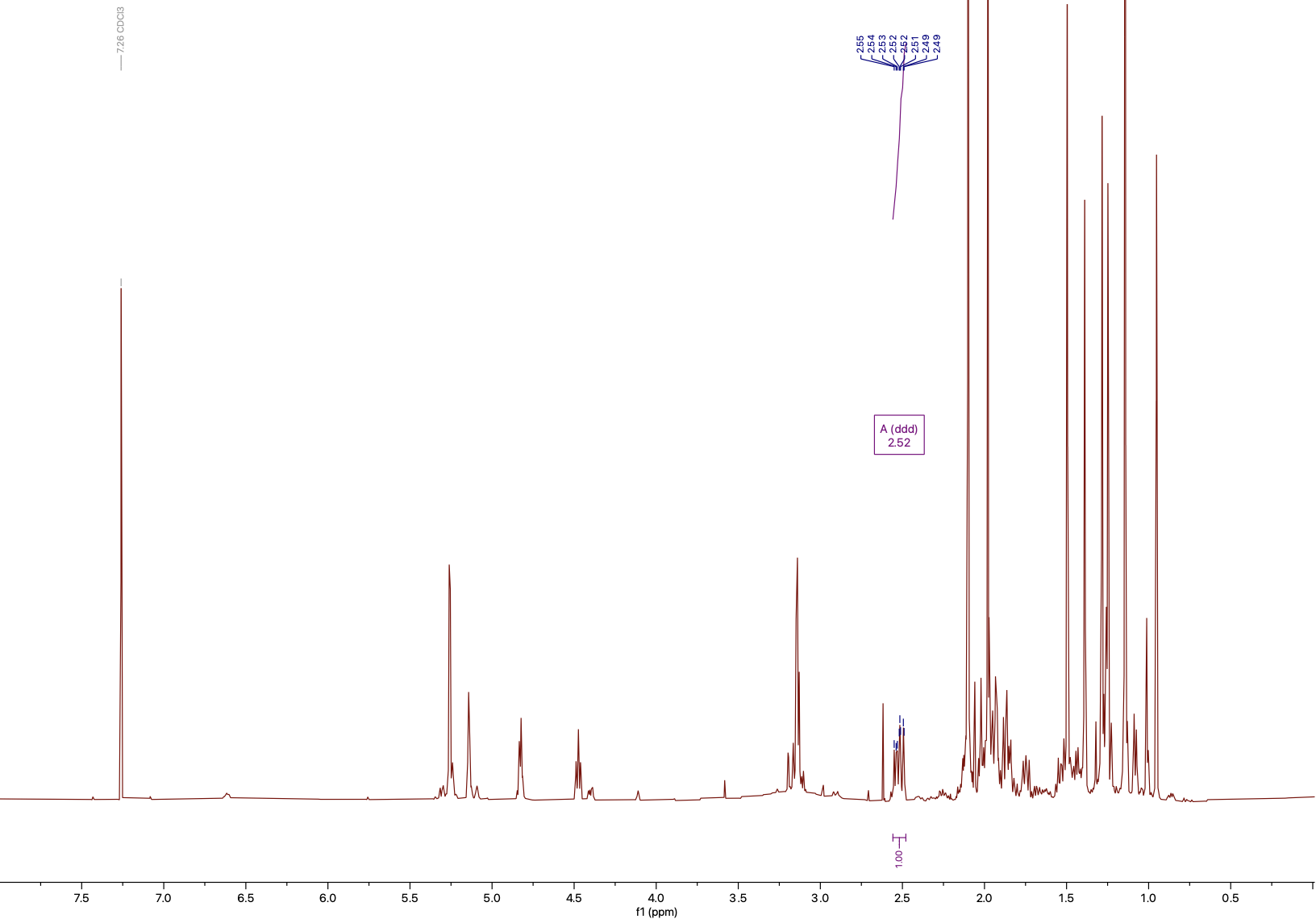


# Figure 32. ^1^H spectrum for (**13’**) (*C. sinensis*).


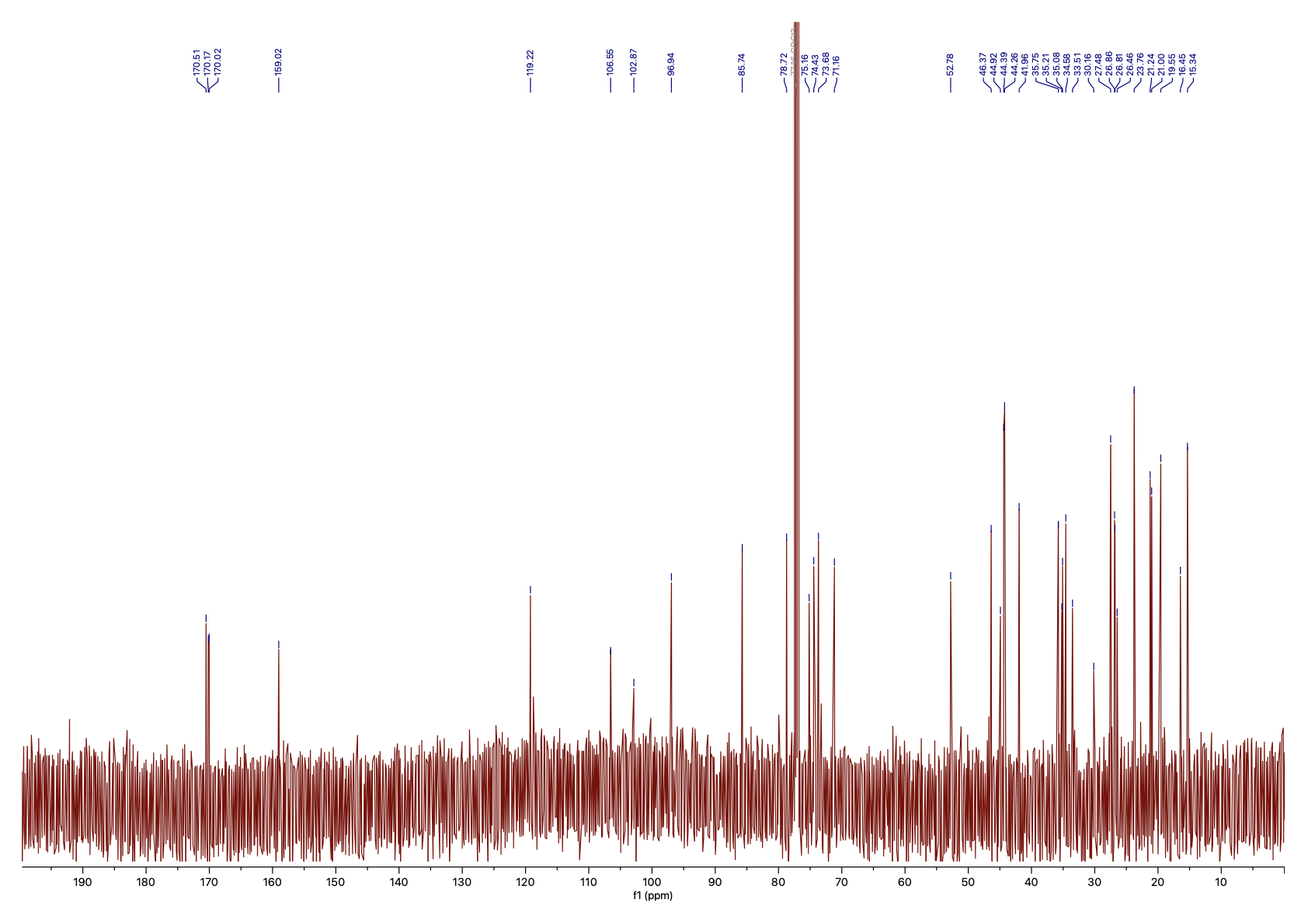


# Figure 33. ^13^C spectrum for (**13’**) (*C. sinensis*).


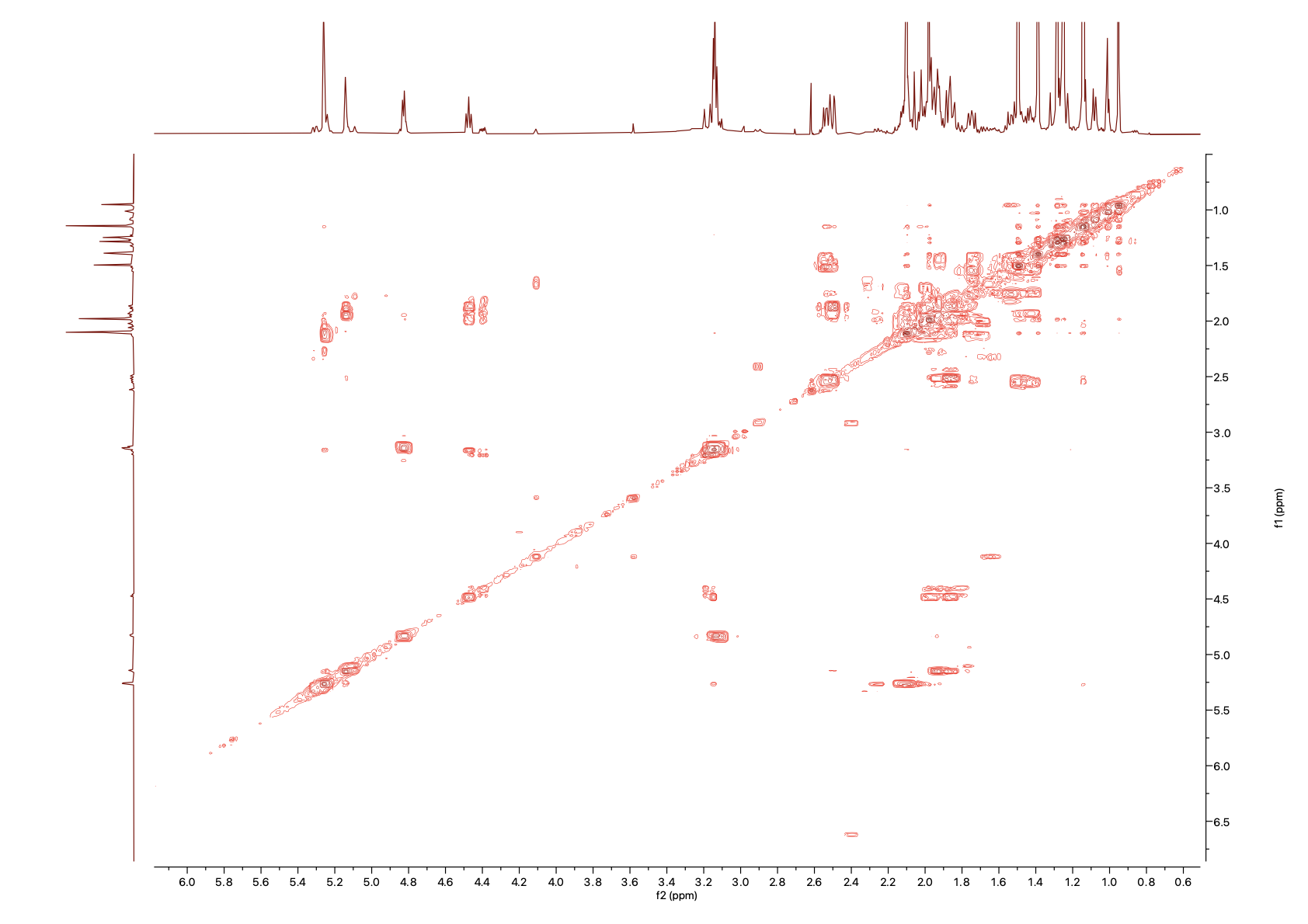


# Figure 34. COSY spectrum for (**13’**) (*C. sinensis*).


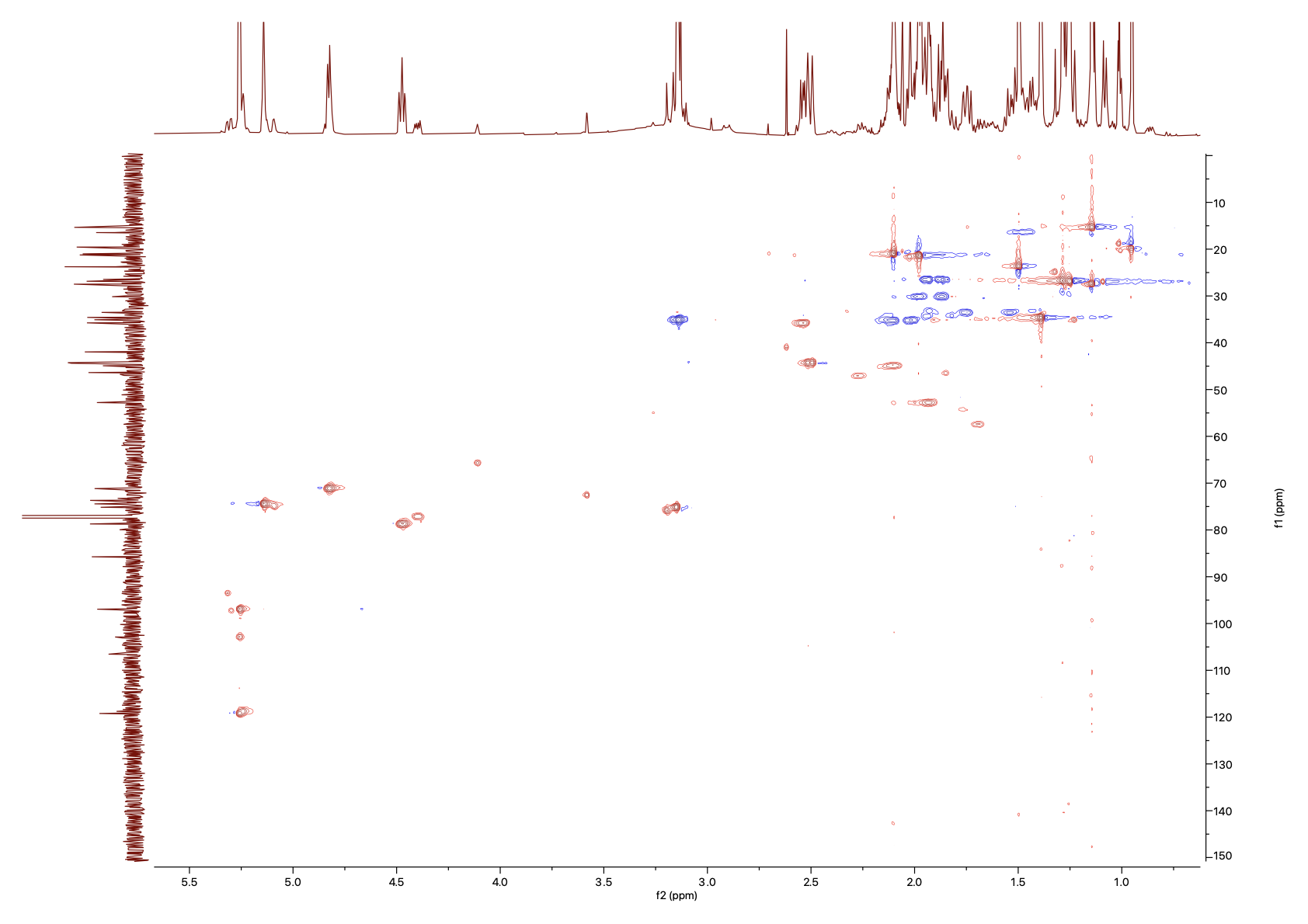


# Figure 35. HSQC spectrum for (**13’**) (*C. sinensis*).


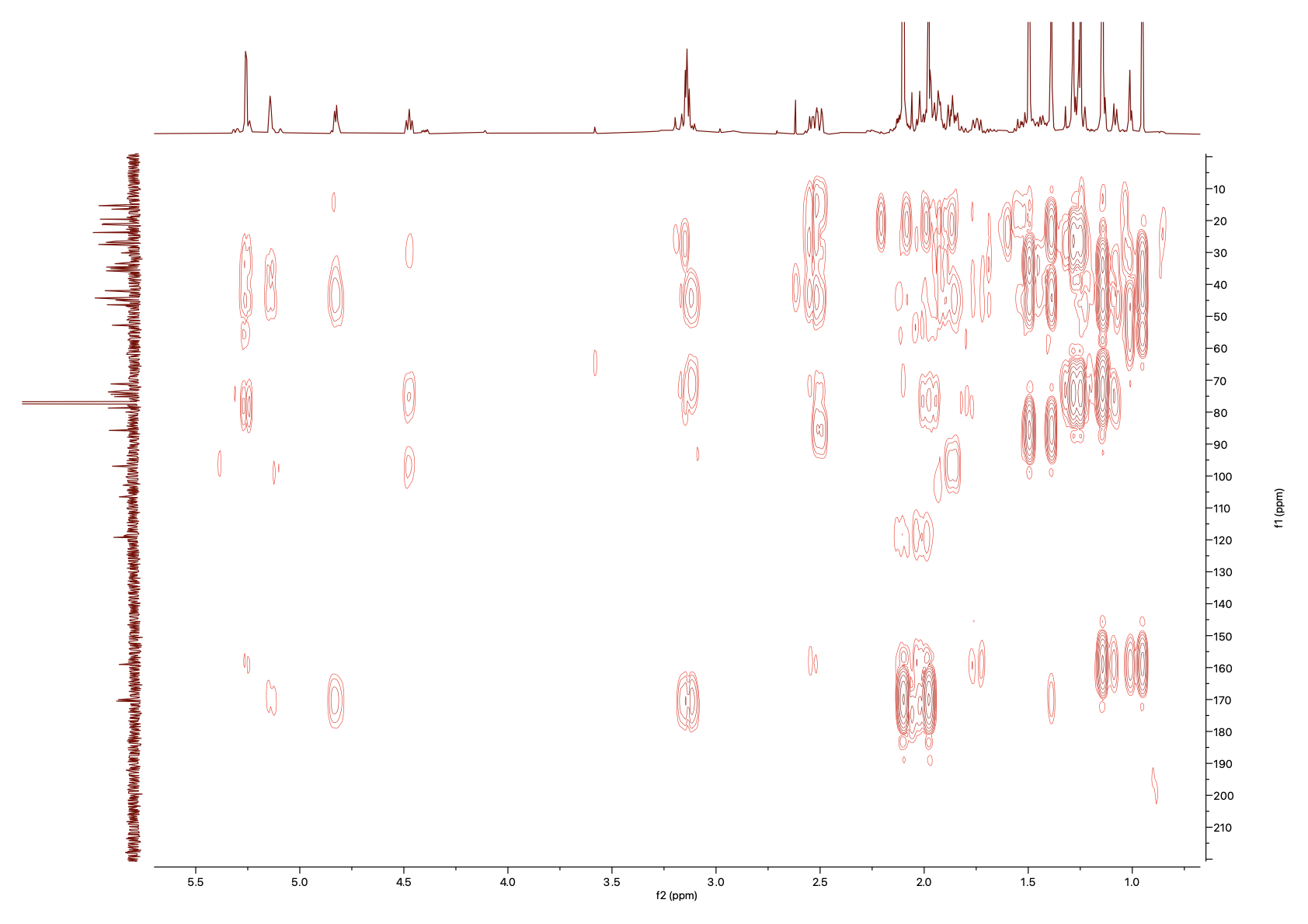


# Figure 36. HMBC spectrum for (**13’**) (*C. sinensis*).


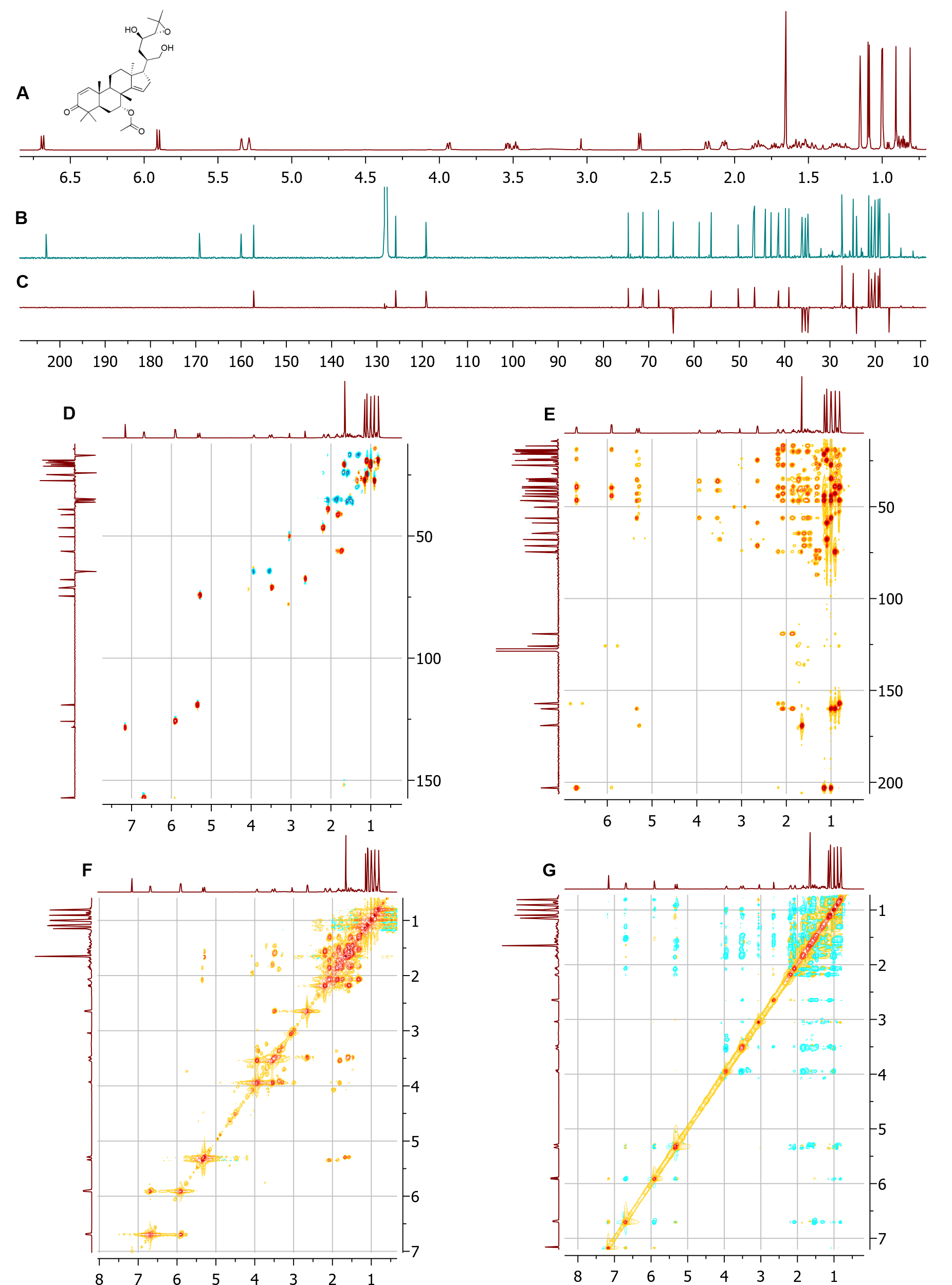


# Figure 37*.* NMR spectra of (14) (*M. azedarach*).

NMR spectra ([Benzene-d_6_], δ (ppm)). (A) ^1^H. (B) ^13^C. (C) DEPT-135. (D) DEPT-edited-HSQC. (E) HMBC. (F) COSY. (G) ROESY.

#


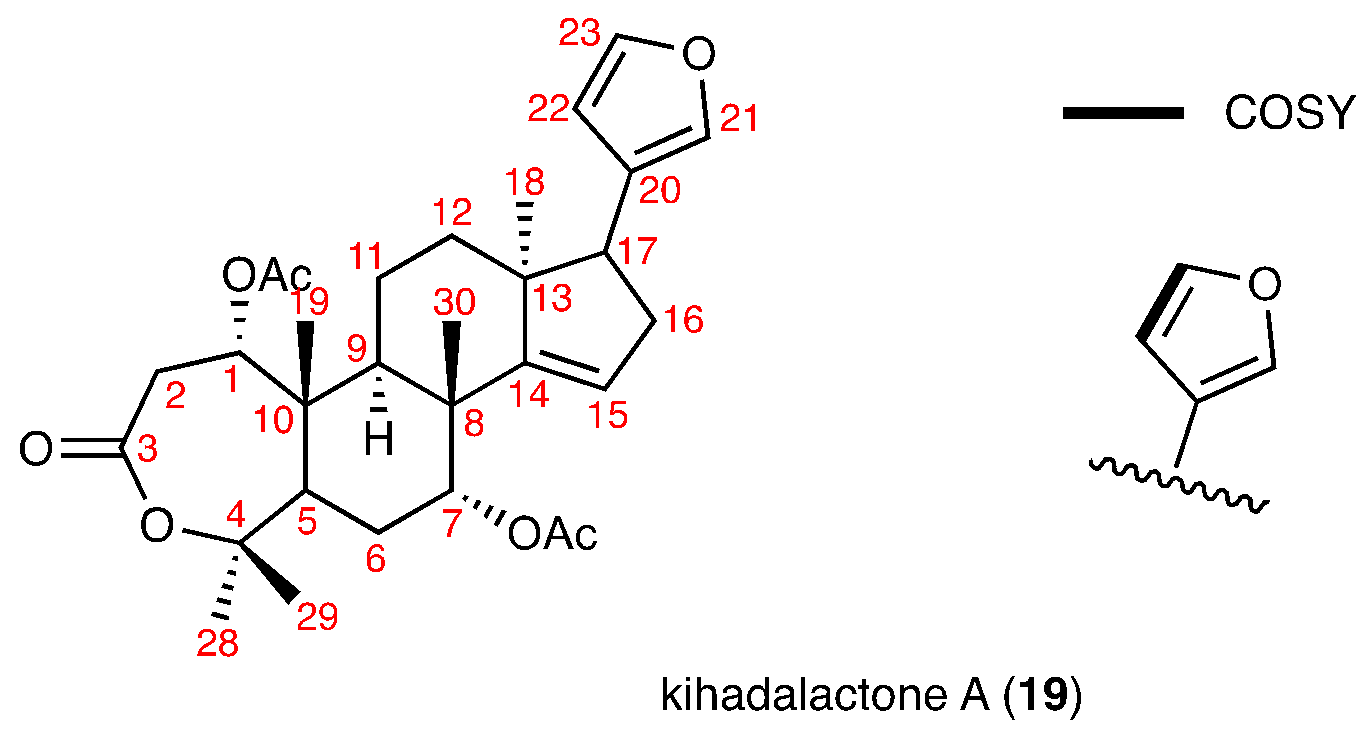


# Figure 38. Partial NMR assignment for kihadalactone A (19) [(*C. sinensis*)](#_heading=h.mza71pomnq27).

Assignment based on NMR spectra ([CDCl_3_], δ (ppm)) listed in Figure 39-40.


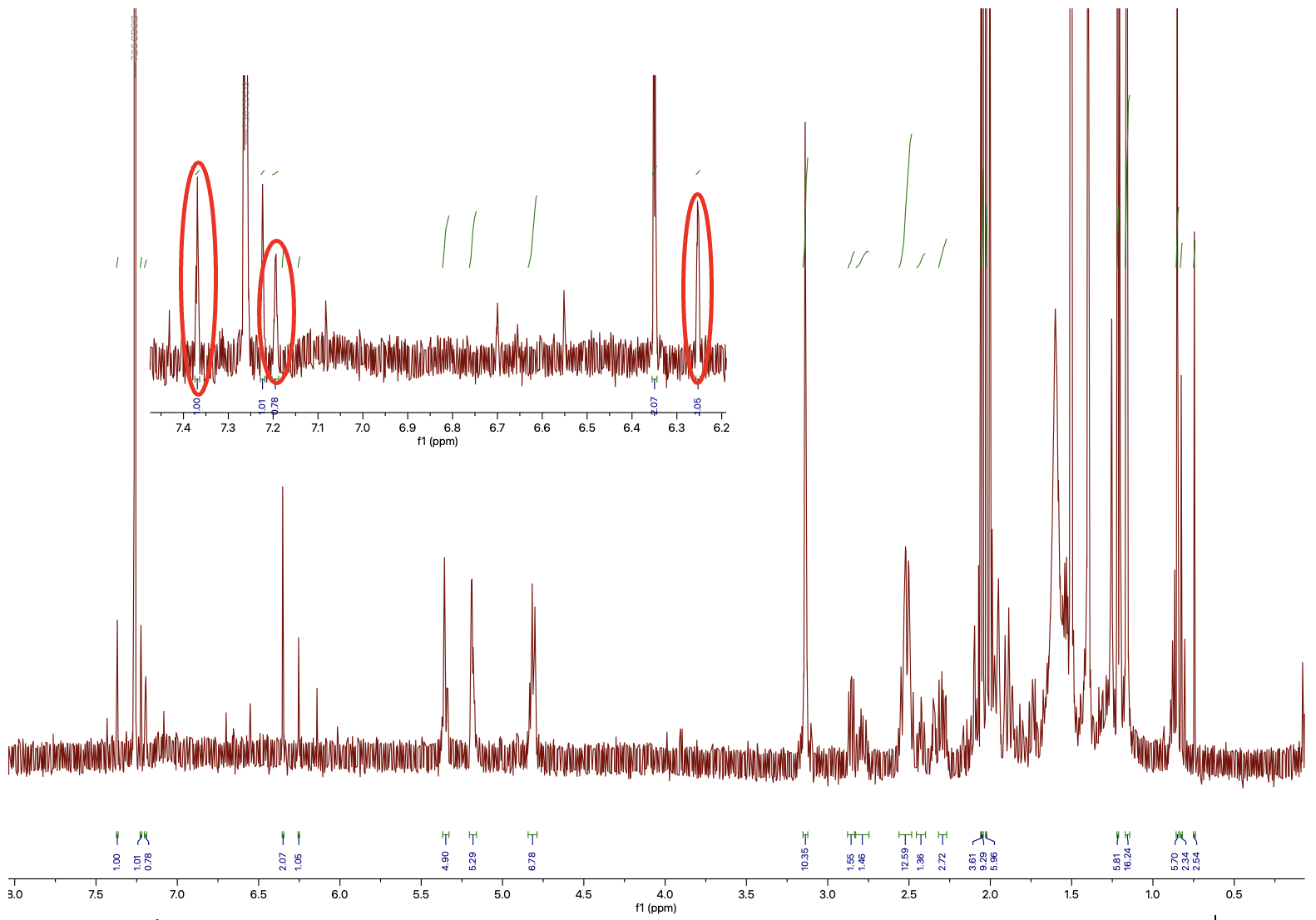


# **Figure 3**9**. 1H spectr**um **for kihadalactone A (19)** [(*C. sinensis*).](#_heading=h.mza71pomnq27)**.**

The sample contained non-furan limonoid impurities such as (**17**). The signature furan protons of (**19**) are circled in red.


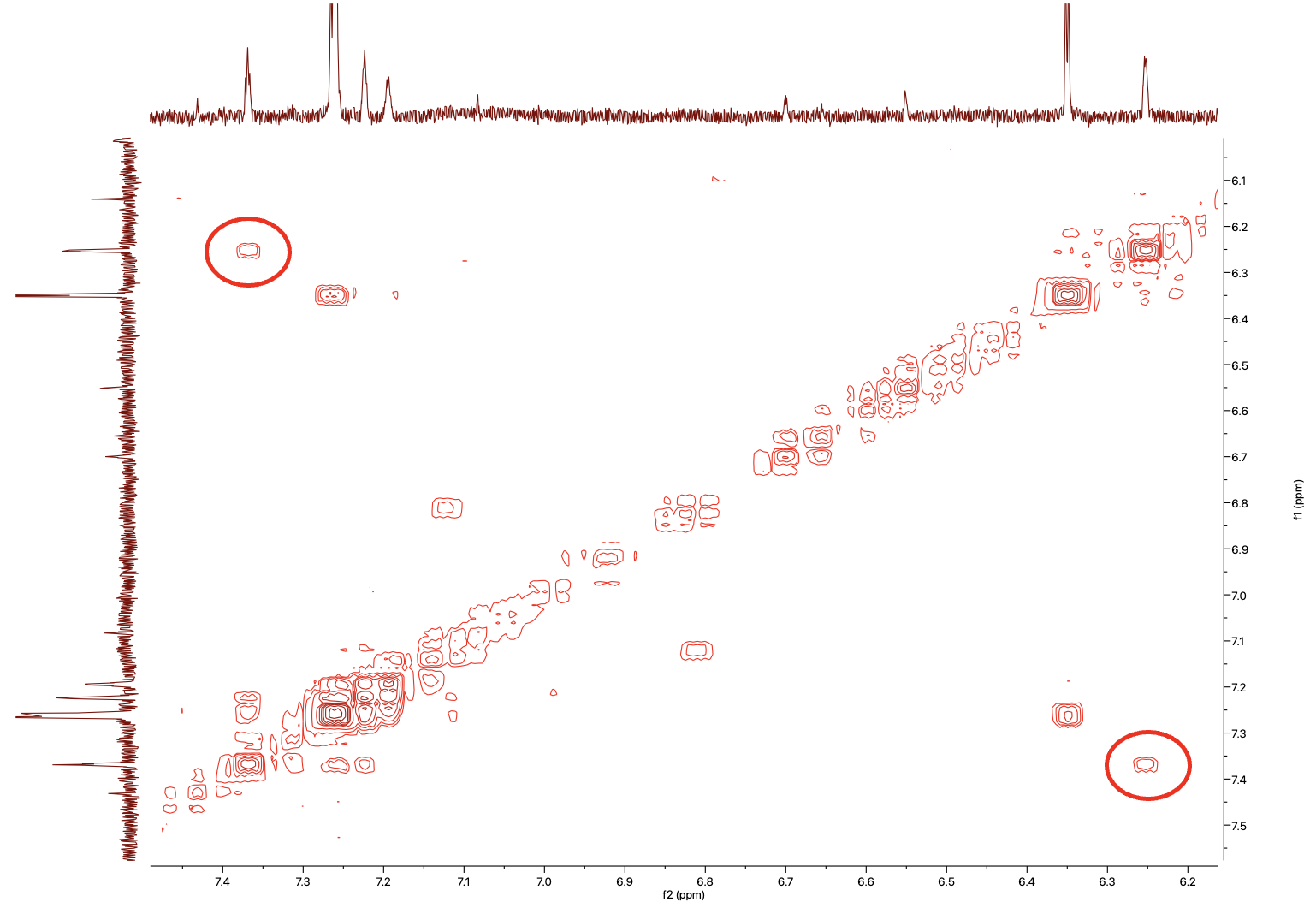


# **Figure 40. COSY spectr**um **for kihadalactone A (19)** [(*C. sinensis*).](#_heading=h.mza71pomnq27)**.**

The sample contained non-furan limonoid impurities such as (**17**). The signature furan proton correlations of (**19**) are circled in red.


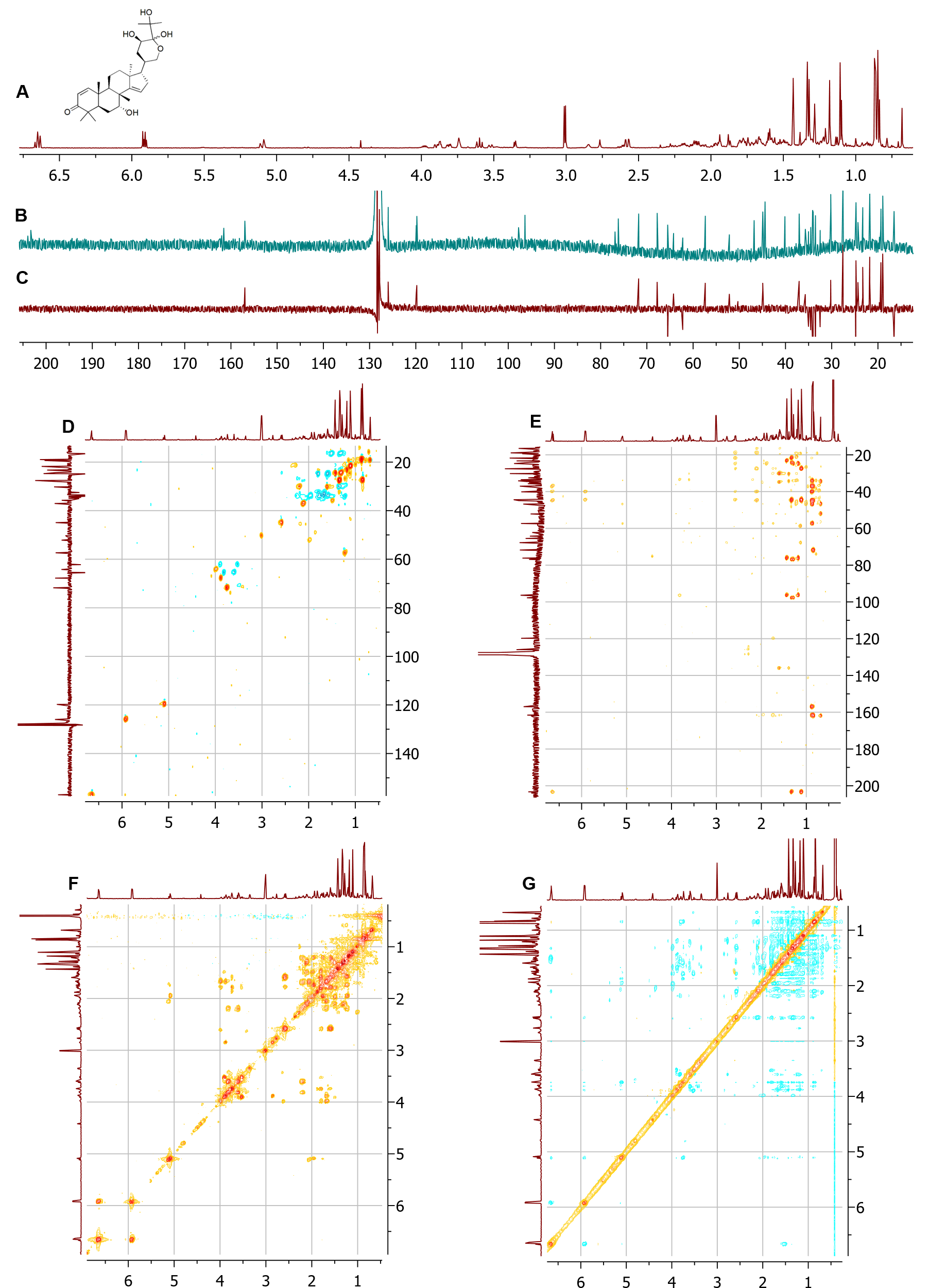


# Figure 41. NMR spectrum of (20) (*M. azedarach).*

NMR spectra of epimeric mixture ([Benzene-d_6_], δ (ppm)). (A) ^1^H. (B) ^13^C. (C) DEPT-135. (D) DEPT-edited-HSQC. (E) HMBC. (F) COSY. (G) ROESY.


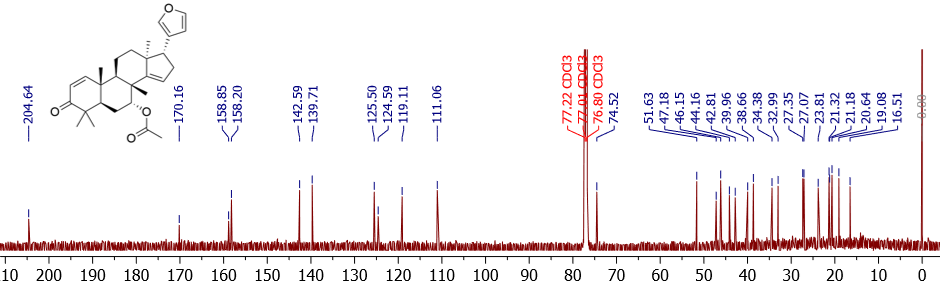


# Figure 42. ^13^C NMR spectrum of analytical standard of azadirone (18).

NMR spectra [CDCl3], δ (ppm) of azadirone isolated from *A. indica*.
